# Sanguinarine Induces Pyroptosis *via* the Hippo Signaling Pathway and Remodels the Tumor Immune Microenvironment in Lung Adenocarcinoma

**DOI:** 10.7150/ijbs.139061

**Published:** 2026-10-02

**Authors:** Jia Yang, Lanfang Li, Yunjian Pan, Wulei Hou, Xiaoyan Wang, Zhengda Li, Wenjie Wang, Yihua Sun, Ping Xu, Lifang Wang, Ying Lu, Zhongqi Wang, Zhenhui Lu, Zuoyun Wang

**Affiliations:** 1 Department of Oncology, Longhua Hospital Shanghai University of Traditional Chinese Medicine, Shanghai 200032, China.; 2 Department of Anatomy, Histology and Embryology, School of Basic Medical Sciences, Shanghai Pudong Hospital, Fudan University, Shanghai 200032, China.; 3 Fudan University Shanghai Cancer Center, Shanghai 200032, China.; 4 Huadong Hospital Affiliated to Fudan University, Shanghai 200040, China.; 5 Institute of Respiratory Diseases, Longhua Hospital Shanghai University of Traditional Chinese Medicine, Shanghai 200032, China.; 6 Bayinguoleng Mongolian Autonomous Prefecture Hygiene School, Xinjiang Province 841000, China.; 7 Department of Biochemistry and Molecular Biology, School of Basic Medical Sciences, Fudan University, Shanghai 200032, China.

**Keywords:** LUAD, Hippo signaling pathway, sanguinarine, pyroptosis, tumor immune microenvironment

## Abstract

Sanguinarine (SAN), a natural alkaloid derived from Papaveraceae plants, is an active component in traditional Chinese medicines. SAN exerts inhibitory effects on tumor cells, but research on its effects on lung adenocarcinoma (LUAD) and the underlying mechanisms remains limited. The Hippo signaling pathway is commonly downregulated in LUAD, making it a key target for therapeutic intervention. Our study reveals that SAN significantly inhibits LUAD. Mechanistically, SAN bound to Large Tumor Suppressor 1 (LATS1) and promoted its phosphorylation, thereby activating the Hippo pathway and pyroptosis in LUAD. Notably, YAP overexpression inhibited pyroptosis and weakened the antitumor effect of SAN, further confirming that SAN exerts its antitumor effects by regulating the Hippo-YAP axis. Findings from both *in vivo* experiments and single-cell RNA sequencing consistently show that SAN remodels the tumor immune microenvironment and inhibits tumor growth. Specifically, we observed significant changes in the tumor microenvironment, including increased CD8^+^ T cell infiltration and a notably reduced number of myofibroblastic cancer-associated fibroblasts. Collectively, our findings establish SAN as a candidate therapeutic agent that exerts antitumor effects through dual modulation of the Hippo-mediated pyroptosis pathway and tumor immunity, providing a mechanistic basis for its potential clinical application in LUAD.

## Introduction

Lung cancer remains a devastating global public health crisis with profound clinical and socioeconomic implications. Lung adenocarcinoma (LUAD) comprises about 40% of all cases and represents one of the major causes of cancer-associated mortality globally. Despite advancements in chemotherapy, targeted therapy, and immunotherapy, its long-term survival rate remains relatively low 1, 2. Beyond its staggering epidemiological impact, LUAD carries a poor prognosis because of late-stage diagnosis, and causes substantial functional impairments, including dyspnea, reduced exercise capacity, and psychological distress, in 40%-80% of patients 3. Moreover, challenges such as therapeutic resistance, and distinct disease patterns among nonsmokers and specific ethnic groups further exacerbate disease severity. Current LUAD therapeutics, including chemotherapy, targeted agents, and immune checkpoint inhibitors, have improved patient prognosis, but they exhibit notable limitations. Specifically, chemotherapy lacks tumor specificity, causing severe off-target toxicity and displaying limited efficacy. Targeted therapy is effective only in patients with specific driver mutations, and drug resistance inevitably arises because of target mutations or alternative pathway activation. The utility of immune checkpoint inhibitors is hampered by low response rates, immune-related adverse events, and difficulty in patient selection 4-6. Despite the availability of the aforementioned modalities for LUAD, their inherent limitations represent an urgent clinical priority.

Sanguinarine (SAN) is a natural compound derived from Papaveraceae plants such as *Chelidonium majus*, and it exhibits potent and versatile antitumor activities across a broad spectrum of cancers. In colorectal cancer (CRC), SAN and its derivatives exert significant anticancer effects through multiple mechanisms: targeting mixed lineage kinase 4, suppressing migration and metastasis by reversing EMT, and inducing oxeiptosis through the H_2_O_2_ dependent KEAP1-PGAM5-AIFM1 signaling axis 7-9.

Additionally, SAN inhibits oral squamous cell carcinoma growth by targeting the PKM2-TFEB axis to impair lysosomal function and block autophagic flux 10. These effects across non-lung cancers are often linked to the regulation of key signaling pathways, including the JAK-STAT, NF-κB, and PI3K-Akt/mTOR pathways, underscoring the multifaceted mode of action of SAN 11. Notably, SAN displays remarkable therapeutic potential in lung cancer, particularly in overcoming treatment resistance and targeting specific subtypes. In non-small cell lung cancer (NSCLC), SAN can induce the release of cytochrome c and activating caspases (caspase-3, caspase-8, caspase-9), while inhibiting the constitutively active JAK-STAT pathway. 12. Critically, SAN effectively overcomes tyrosine kinase inhibitor (TKI) resistance in NSCLC. It selectively kills TKI-resistant cells by specifically upregulating NADPH oxidase 3 (NOX3) to induce reactive oxygen species generation, leading to redox imbalance between NOX3 and methionine reductase A, subsequent oxidation of EGFR at Met790, and eventual proteasomal degradation of mutant EGFR 12, 13. Collectively, these results suggest that SAN holds promise as a candidate for novel anticancer drug development, with well-defined molecular targets and broad applicability across diverse cancer types. Despite the therapeutic potential of SAN, its antitumor effects and underlying mechanisms in LUAD have yet to be fully elucidated. Further research is urgently needed to provide a solid theoretical foundation for its clinical application.

Large Tumor Suppressor 1 (LATS1) constitutes a core kinase component of the Hippo signaling pathway. Functioning as a “gatekeeper” downstream of the cascade, LATS1 transduces signals from the upstream MST1/2 kinases and exerts its effects by directly phosphorylating and thereby inactivating the transcriptional co-activators YAP and TAZ, culminating in the inhibition of cell proliferation and induction of apoptosis. LATS1 plays an essential anti-tumor role in the control of organ size, maintenance of tissue homeostasis, and neoplastic transformation. Diminished expression or loss of function of LATS1 represents a major driving force underlying the malignant progression of multiple human malignancies, including mesothelioma and breast cancer, underscoring its considerable potential as a therapeutic target in the context of precision oncology 14. YAP, a core effector of the Hippo signaling pathway, was initially identified as a binding partner of Yes tyrosine kinase, but it has subsequently been recognized as a critical regulator of cell proliferation, differentiation, apoptosis, and organ size 15, 16. As a key downstream effector of Hippo signaling, YAP activity directly influences organ growth. Upon Hippo inhibition, YAP moves to the nucleus and enhances TEAD-dependent transcription, enhancing proliferation, suppressing apoptosis, and ultimately leading to organ enlargement. YAP also plays context-dependent roles in cell differentiation. Specifically, high YAP activity supports self-renewal by preserving an undifferentiated state, whereas YAP inhibition promotes lineage commitment and differentiation 17.

Despite the established functions of this signaling axis, the relationship between YAP and pyroptosis remains poorly understood. Mediated by inflammasomes and executed by gasdermins, pyroptosis is a type of programmed cell death that features membrane perforation, content release, and robust inflammatory responses. Its initiation involves a complex cascade of signal reactions: when cells detect pathogens or damage-associated molecular patterns (DAMPs), the NLRP3 inflammasome is activated and recruits ASC and caspase-1 to assemble into a multiprotein complex. The activated caspase-1 cleaves gasdermin proteins (e.g., GSDMD), liberating the N-terminal fragment that forms pores in the membrane. Additionally, cellular factors such as metabolic status and redox balance modulate pyroptosis. For instance, oxidative stress can influence this process through related signaling pathways 18. Although several studies have implicated the Hippo pathway in pyroptosis regulation 19, the direct regulatory link between YAP and pyroptosis has not been clearly elucidated. Identifying new YAP-dependent tumor-suppressive mechanisms holds great promise for the discovery of novel targeted therapies, facilitating the development of more precise clinical interventions in cancer.

Physiologically, pyroptosis contributes to embryonic development and tissue repair, whereas pathologically, it has been implicated in inflammatory and autoimmune diseases. In early tumorigenesis, pyroptosis can serve as a defense mechanism to eliminate malignant cells. Given its broad involvement in disease, targeting the pyroptosis pathway has emerged as a promising therapeutic strategy, making it a focal point in biomedical research. For NSCLC treatment, chemotherapeutic agents such as cisplatin and paclitaxel differentially induce pyroptosis 20. Natural compounds such as cucurbitacin B (CuB) further expand pyroptosis-inducing strategies by activating the TLR4-NLRP3-GSDMD pathway 21. Moreover, nanoplatform-based co-delivery systems synergistically amplify pyroptosis (*via* the NLRP3-GSDMD-caspase-1 axis) and ferroptosis, thereby further enhancing the therapeutic efficiency of the disease 22.

Moreover, the therapeutic potential of pyroptosis activation in lung cancer has been shown to be closely linked to its capacity to reshape the tumor immune microenvironment (TIME). Robust and acute pyroptosis triggers tumor cell lysis and the release of DAMPs, including HMGB1 and IL-1β/IL-18, which in turn recruit dendritic cells and stimulate CD8⁺ T cells, thus transforming "cold" tumors into "hot" ones. 22-24. Pyroptosis-related molecules serve as potential biomarkers for predicting chemotherapy sensitivity, whereas targeted inhibitors (e.g., PGAM1 inhibitor HKB99) or activators (e.g., CuB) can be used to fine-tune pyroptosis and reverse TIME dysfunction 21, 25, 26.

A growing body of research indicates that the caspase-1 and GSDMD represent viable therapeutic targets for lung cancer. ROS trigger pyroptosis via the ROS-NLRP3-caspase-1-GSDMD signaling cascade, which in turn potentiates the antitumor efficacy in lung cancer therapy 27, 28. Several potential therapeutics targeting the pyroptosis pathway are currently under development. Inhibitors such as MCC950 specifically block NLRP3 inflammasome activation, thereby suppressing pyroptosis, whereas antibody-based drugs targeting gasdermins are being designed to modulate their pore-forming activity. Although these agents hold promise for treating pyroptosis-related diseases, they remain in preclinical development. As an emerging mode of inflammatory cell death, pyroptosis offers novel perspectives on disease mechanisms. Ongoing studies should aim to clarify the molecular pathways through which pyroptosis operates in specific disease settings, thereby promoting the clinical translation of these discoveries.

Our study demonstrated that SAN exerts antitumor effects through dual mechanisms. Mechanistically, SAN bound to LATS1, promoting its phosphorylation and subsequent YAP nuclear export and degradation, which triggers pyroptosis and inhibits LUAD progression. *In vivo*, SAN further remodels the tumor microenvironment to enhance its therapeutic efficacy. These findings reveal a novel mechanism by which SAN suppresses LUAD and expand the known functions of YAP to include pyroptosis regulation. This work provides a mechanistic foundation and theoretical rationale for the potential clinical application of SAN in LUAD treatment.

## Materials and Methods

### Cell lines and tissue samples

A549 (ATCC-CRM-CCL-185) cells were cultured in high-glucose DMEM (Gibco). H1299 (ATCC-CRL-5803), H1437(ATCC-CRL-5872) and PC9 (RRID: CVCL_D7FJ) cells were cultured in RPMI-1640 medium (Gibco). BEAS-2B (ATCC-CRL-3588) cells were cultured in Airway Epithelial Cell Basal Medium (Gibco). All cells were cultured under standard conditions, with 10% FBS (ExCell Bio). The cells were placed in a 37°C, 5% CO₂ incubator. Cells in the exponential growth phase were used for the experiments. Human tumor tissues were collected from Fudan University Shanghai Cancer Center. Ethics approval for this study was obtained from Medical Ethics Committee of Affiliated Hospital of Fudan University at Affiliated Hospital of Fudan University (approval number: 20240229-003). All participants were provided with information regarding the study and gave their written informed consent prior to participation. This study was conducted in compliance with the Declaration of Helsinki and all applicable ethical guidelines.

### *Kras^ LSL-G12D/+^; Trp53^fl/fl^* mouse models

*Kras^ LSL-G12D/+^; Trp53^fl/fl^* mice were infected by intranasally delivering a 63.5 μL suspension of 2 × 10^7^ PFU Adeno-Cre. After 2 weeks, the mice were intraperitoneally injected with sodium chloride solution or SAN every 3 days for the remainder of the experiment. Tumor sizes and numbers in the lungs were measured by IHC after 2 months. Ethics approval for this study was obtained from Laboratory Animal Center Fudan University at Fudan University (approval number: 2021-078).

### Lung cancer organoid culture

Surgically resected primary tumors were collected from patients with LUAD. For the cultivation of organoids, the tumor tissues are cut into 5-millimeter diameter blocks and washed with PBS. Subsequently, at 37°C, the tumor blocks are treated gently with a shaking motion using Advanced DMEMF12 (GIBCO) medium and collagenase XI (Sigma) for 40 minutes. Cells were counted and resuspended in growth factor-reduced basement membrane extract (BME, R&D), plated in 24-well tissue culture plates as BME domes and maintained at 37℃ with 5% CO_2_ with medium overlaying the BME dome. Organoid growth was monitored weekly.

### CCK-8 and colony formation assays

For the CCK-8 and cell aggregation experiments, human lung cancer cell lines (A549 and H1299) as well as normal lung epithelial cells (BEAS-2B) were plated into 96-well plates at a seeding density of 1 × 10^4^ cells/well. Viability was quantified spectrophotometrically (OD450) after incubating cells with CCK-8 reagent (Yeasen Biotechnology) per the manufacturer's guidelines. Clonogenic potential was assessed after fixing (4% paraformaldehyde [PFA]) and staining (crystal violet) 2-week cultures in six-well plates, with colonies counted using ImageJ software. For the soft agar colony formation assay, a 0.8% agarose solution was used as the bottom layer, and a 0.5% agarose solution containing 1 × 10⁴ cells/mL A549 cells and the corresponding concentrations of SAN was used as the upper layer. After 30 days of culture, colonies with a diameter exceeding 50 μm were counted.

### Migration assays

A549 and H1299 cells were plated in six-well plates and grown to 80% confluence. A linear scratch was made uniformly across the monolayer using a 10 μL pipette tip. Cells were then cultured in 5% FBS medium containing SAN (0, 1.5, or 3 μM) or the vehicle control. Images of the scratches were taken at 0, 12, and 24 h post-wounding under an inverted microscope, and the percentage of wound closure was calculated using ImageJ with the formula: [(wound width at 0 h - wound width at indicated time) / wound width at 0 h] × 100%. In the transwell chamber assay, 1 × 10⁵ A549 or H1299 cells suspended in serum-free medium were added to the upper inserts, and the lower chambers were filled with 10% FBS medium containing SAN. Following a 48-h incubation, cells remaining on the upper membrane surface were wiped off. The migrated cells on the lower side were fixed with 4% PFA for 20 min, stained with crystal violet, and counted under a microscope.

### Immunoblotting

Cells and tumor tissues were lysed in RIPA buffer on ice to obtain total protein extracts. The lysates were subjected to SDS-PAGE (10%) to separate proteins according to size, followed by electroblotting onto PVDF membranes. The membranes were then blocked with 5% milk for 1 h and probed with primary antibodies diluted in blocking buffer at 4°C overnight. Thereafter, the blots were incubated with HRP-linked secondary antibodies for 2 h. Then, the bands on the membrane were visualized under a chemiluminescence imaging system using an enhanced chemiluminescence kit. GAPDH and β-actin were used as loading controls. Western blots were probed with mouse anti-GAPDH (ABclonal; RRID: AC003), mouse anti-β-actin (ABclonal; RRID: AC004), rabbit anti-LATS1 (Cell Signaling Technology; RRID: 3477S), rabbit anti-pLATS1 (Cell Signaling Technology; RRID: 9157S), rabbit anti-MOB1 (Cell Signaling Technology; RRID: 13730S), rabbit anti-p-MOB1 (Cell Signaling Technology; RRID: 8699S), rabbit anti-YAP (Cell Signaling Technology; RRID: 14074S), rabbit anti-p-YAP (Cell Signaling Technology; RRID: 4911S), rabbit anti-caspase-1 (absin; RRID: abs128793), rabbit anti-cleaved caspase-1 (ABclonal; RRID: A23429), rabbit anti-GSDMD (Cell Signaling Technology; RRID: 39754T), or rabbit anti- cleaved GSDMD antibody (ABclonal; RRID: A24059).

### IHC and IF staining

Tumor tissues from Kras ^LSL-G12D/+^; Trp53^fl/fl^ mice were fixed in 4% PFA, embedded in paraffin, and sectioned. The sections were deparaffinized, rehydrated, and subjected to antigen retrieval in citrate buffer at 95°C for 30 minutes. Incubating with goat serum for 1 hour, the sections were incubated with the primary antibodies against Ki-67, GSDMD, GSDMD-N, caspase-1, cleaved-caspase-1, caspase-3, and YAP (1:200 dilution). After incubation with enzyme-conjugated secondary antibody, the signals were visualized using 3,3'-diaminobenzidine substrate. Following counterstaining with hematoxylin, dehydration, clearing, and mounting, the sections were imaged under a microscope, and positive staining was quantified using ImageJ software.

### Plasmid construction and transfection

To validate the regulatory role of Hippo pathway key molecules (YAP and LATS1) in SAN induced lung cancer cell pyroptosis, recombinant plasmids were constructed. The full-length coding sequence of human *YAP* (GenBank: NM_001130145.1) and human *LATS1* (GenBank: NM_ 001270519.2) were amplified from A549 cell cDNA and cloned into a pcDNA3.1-HA (RRID: Addgene_128034) vector to generate overexpression plasmids. All the target fragments and vectors were ligated with T4 DNA ligase, and then transfected into *Escherichia coli* DH5α. The positive clones were verified by Sanger sequencing. We constructed the LATS1 triple-mutant plasmid via homologous recombination. Specifically, DNA fragments carrying the mutation sites and flanked by homology arms were first generated. The vector was then linearized by digestion with appropriate restriction enzymes. Subsequently, the mutated DNA fragments and the linearized vector were subjected to homologous recombination using recombinase. Finally, the complete plasmids with correct site mutations were verified by Sanger sequencing. High-purity endotoxin-free plasmids (OD260/OD280: 1.8-2.0) were prepared using EndoFree Mini Plasmid kits. The overexpression/knockdown efficiency was confirmed by qPCR and immunoblotting after transfecting KP1 and H1299 cells.

### RNA isolation and RT-qPCR

RNA was extracted from cells using TRIzol, and cDNA synthesis was performed with PrimeScript™ RT Master Mix. Real-time PCR was conducted using TB Green™ Premix Ex Taq™ II (Takara). Gene expression levels were quantified relative to β-actin via the 2^⁻ΔΔCt^ method^29^.

### Receptor model and molecular docking (MOE)

A template-based homology model of human LATS1 (residues 662-1,062; UniProt accession O95835) was retrieved from the “Structure” section of UniProt as a SWISS-MODEL entry (model identifier: O95835_662-1062:sn23.1.A, chain A). The model was prepared by adding hydrogen atoms and assigning protonation states under physiological conditions, followed by restrained energy minimization to alleviate local steric clashes while preserving the overall fold. Molecular docking of SAN was performed using MOE with the modeled LATS1 structure as the receptor. The binding site was defined according to the predicted pocket surrounding the key interacting residues observed in the docking pose, and the top-ranked pose was selected according to the docking score and reasonable interaction geometry. As the receptor is a homology model, the interaction pattern is described as predicted docking.

### Statistical analysis

All data in this study are represented as the mean ± mean standard error. GraphPad Prism (version 8.3.0) and Image J were used to analyze the results and draw charts. Between-group differences were analyzed by Student's t-test. One-way ANOVA with Bonferroni correction was used for normally distributed data when comparing more than two groups, whereas the Kruskal-Wallis test with Dunn's post-hoc test was employed for non-normal data. Statistical significance was defined as P < 0.05.

## Results

### SAN inhibits the proliferation, invasion, and migration of LUAD cells

The significant effects of SAN on breast and CRC cells have been confirmed. To characterize the functional effects of SAN on LUAD progression, we tested its effects on LUAD cell proliferation, invasion, and migration. At a concentration of 1.25 μM, SAN treatment significantly inhibited the proliferation of A549 and H1299 LUAD cells versus the vehicle control, but it did not significantly affect the proliferation of BEAS-2B normal lung epithelial cells (Figure 1A). Ki-67, a cell proliferation marker 30 exhibited similar expression changes upon SAN treatment (Figure S5C, D). Likewise, SAN significantly inhibited the clonal formation, invasion, and migration of A549 and H1299 cells (Figures 1B-E, S1, S2). Furthermore, SAN upregulated the expression of the E-cadherin and downregulated the expression of mesenchymal markers in a concentration-dependent manner, indicating that SAN reverses EMT, thereby potentially suppressing LUAD metastasis (Figure S3). Existing studies have shown that SAN has excellent security features 31, 32. Meanwhile, we measured the serum levels of aspartate aminotransferase and alanine aminotransferase in SAN-treated and control mice, and statistical analysis revealed no significant differences between the two groups (Figure S4). We further assessed structural differences in the heart, liver, spleen, lung, and kidney tissues of mice in the two groups. HE staining revealed no significant histological differences between the two groups (Figure S4). We utilized *in vivo* mouse models to test the functional role of SAN in LUAD growth. Treatment with SAN intraperitoneally every 3 days from day 9 after subcutaneous KP1 cell inoculation significantly inhibited tumor growth compared with the effects of the vehicle controls, confirming the antitumor activity of SAN (Figure 1F-H). The comparable body weight profiles of SAN-treated and control mice further supported the safety and tolerability of the drug *in vivo* (Figure S9A). Furthermore, oxygen probe measurements revealed that SAN significantly boosts ROS production (Figure 1I-J). Collectively, these data position SAN as a promising therapeutic candidate for LUAD, demonstrating its potent tumor-suppressive effects.

### SAN induces pyroptosis in LUAD cells

Accumulating evidence has established a causal link between ROS accumulation and the induction of pyroptosis 33-35. As previously demonstrated, SAN treatment led to a significant increase in cellular ROS production (Figure S6), prompting further investigation into its downstream effects. Treatment of LUAD cells (A549, H1299, KP1, and PC-9) with SAN induced progressive cell swelling and, in some cases, rupture. These morphological changes are characteristic of a lytic form of cell death (Figures 2A-B, S5A), which led us to hypothesize that SAN triggers cell death via pyroptosis. Pyroptosis is a unique form of programmed cell death driven by a specific mechanism: inflammasome activation triggers caspase-1, which cleaves gasdermins. The liberated N-terminal fragments form membrane pores, disrupting osmotic balance and leading to cell lysis and inflammation 23, 36-38. Increased protein expression of cleaved caspase-1 and GSDMD N-terminal fragment (GSDMD-N, the active form of GSDMD) following SAN treatment were confirmed by western blotting and immunofluorescence (IF) staining of cell lysate collected from KP1 and H1299 cells (Figure 2C-J). Consistently, SAN treatment elevated caspase-1 expression in KP1 cells (Figure S7A) and GSDMD expression in A549 and H1299 cells (Figures S5B, S7B). These findings indicate that SAN triggers pyroptosis in LUAD cells by promoting ROS accumulation. This mechanism, in turn, contributes significantly to SAN's ability to inhibit LUAD progression.

### SAN activates Hippo signaling and induces pyroptosis by promoting YAP degradation

To investigate the molecular mechanisms underlying SAN's effects, we performed transcriptome sequencing using SAN-treated A549 cells. The Hippo pathway, a key regulator of LUAD cell proliferation 39-41, underwent significant changes following SAN treatment based on Gene Ontology and Enriched Kyoto Encyclopedia of Genes and Genomes pathway analyses (Figure 3A). Differential gene expression analysis revealed significant changes in *YAP1* and *LATS1* expression following SAN treatment (Figure 3B). We further explored whether SAN directly targets the Hippo pathway by molecular docking analysis. Docking in Molecular Operating Environment (MOE) suggested that SAN and LATS1 have the strongest binding affinity (Figure S7C), with SAN adopting a planar pose within a hydrophobic cleft of LATS1 (662-1,062). In the predicted complex, the cationic center of SAN is oriented toward Asp789, consistent with a prominent electrostatic anchoring interaction that helps position the ligand. Notably, Ile713 and Leu711 form H-π conjugations with the aromatic π surface of SAN (i.e., CH-π/alkyl-π contacts contributed by aliphatic C-H groups), providing additional stabilization of the bound conformation. The surrounding residues, including Gly712, Asp783. Ala732, Met782, Leu835, Tyr784, and Asp832, further delineate the pocket through van der Waals packing and shape complementarity. Collectively, these contacts support a binding mode in which electrostatic anchoring at Asp789 and H-π conjugation-driven hydrophobic stabilization around the aromatic core cooperate to retain SAN in the LATS1 pocket (Figure 3C).

Additionally, western blotting revealed that SAN treatment induced LATS1 phosphorylation in A549 and H1299 cells (Figure 3D), providing evidence that SAN directly engages the Hippo signaling pathway. We further generated LATS1 mutant plasmids harboring point mutations (L711A, I713A, and D789A). In the presence of these mutants, the SAN-mediated alterations in the Hippo signaling pathway and pyroptosis were markedly attenuated (Figures S7D). To further elucidate the mechanism, we examined Hippo pathway components in SAN-treated A549 and H1299 cells at both the transcriptional and protein levels. RT-qPCR and western blotting revealed that SAN concentration-dependently increased LATS1 and YAP phosphorylation. Consistently, total YAP protein expression was markedly reduced, indicating that SAN promotes YAP degradation downstream of Hippo pathway activation. In addition, we observed upregulation of the tumor suppressor gene *VGLL4*, which further confirmed the tumor-suppressing function of SAN (Figures 3E-F, S8E-F). IF staining analysis further demonstrated that SAN treatment induced YAP cytoplasmic retention in A549 cells, consistent with LATS1-induced YAP phosphorylation and subsequent nuclear export for degradation (Figure 3G). Furthermore, we discovered that YAP exists in the form of condensate outside the cell nucleus under high SAN concentrations. A similar pattern was observed in KP1 and H1299 cells, confirming the consistency of this effect (Figure S8A-B).

Given the established role of the Hippo pathway in regulating pyroptosis 19, 42, 43, we hypothesized that YAP, a key downstream effector, might mediate SAN-induced pyroptosis. To determine whether YAP directly regulates pyroptosis, we used siRNA to silence *YAP* in KP1 cells. Western blotting revealed that YAP silencing alone was sufficient to induce pyroptosis, as evidenced by increased GSDMD-N expression (Figure 3H). To determine whether YAP degradation is required for SAN-induced pyroptosis, we utilized a YAP mutant (YAP^5SA^) that resists Hippo-mediated degradation. Overexpression of this mutant in H1299 cells markedly attenuated SAN-induced pyroptotic markers (Figure 3I), demonstrating that loss-of-YAP function is a critical downstream event. Subsequently, we employed two complementary pharmacological approaches. Blocking upstream LATS kinase using NIBR-LTSi prevented YAP phosphorylation and degradation, leading to YAP accumulation and suppressing GSDMD cleavage. Conversely, directly inhibiting YAP activity using verteporfin promoted GSDMD cleavage, indicating that YAP functions as a negative regulator of pyroptosis in lung cancer cells (Figure S8C-D).

These results demonstrate that SAN binds to LATS1 to promote its phosphorylation, leading to YAP nuclear export and degradation, and this cascade ultimately induces pyroptosis and suppresses LUAD progression.

### SAN elicits Hippo pathway-mediated pyroptosis to restrict LUAD progression *in vivo*

To verify the mechanism of SAN in LUAD, we employed an *in vivo* mouse model. Lung tumorigenesis was initiated in *Kras^ LSL-G12D/+^; Trp53^fl/fl^* (KP) mice *via* intranasal delivery of Cre-expressing adenovirus 44. After 2 weeks, mice received intraperitoneal injections of SAN every 3 days until the experimental endpoint (day 80 post-instillation), at which time they were euthanized and tissues were harvested (Figure 4A). Body weight, a key indicator of systemic toxicity, did not significantly differ between the SAN-treated and control groups, confirming the safety of SAN administration (Figure S9B). SAN significantly reduced the *in vivo* tumor burden, as evidenced by a marked decrease in the number of surface tumor nodules compared with the number in control mice (Figure 4B). HE staining demonstrated that SAN treatment induced notable histological improvements, with tumor cells displaying regular arrangement and clear boundaries with adjacent tissues, in contrast to the disordered, invasive growth observed in control tumors. This histological evidence was corroborated by quantitative data revealing significant reductions in both the tumor number and burden in SAN-treated mice. Collectively, these results confirmed the *in vivo* efficacy of SAN in inhibiting LUAD (Figures 4C, S9C).

To further characterize the* in vivo* effects of SAN at the cellular level, we performed single-cell sequencing on primary lung tumors from treated and control mice (Figure 4D). Single-cell analysis of lung tumors from control and SAN-treated mice revealed 12 distinct cell lineages (Figure 4E). Within the epithelial cell cluster, SAN treatment significantly reduced the fraction of cycling cells, demonstrating that SAN inhibits epithelial cell proliferation, contributing to its antitumor efficacy (Figures 4F-G, Figure S10D-E). Single-cell transcriptomic analysis of epithelial cells revealed that SAN treatment significantly altered the expression of Hippo pathway components, including* Lats1* and *Yap* (Figure 4H). Consistently, differential gene enrichment analysis confirmed substantial modulation of the Hippo signaling pathway upon SAN treatment (Figure 4I). Moreover, pyroptosis-related genes, particularly *Gsdmd*, were markedly upregulated in SAN-treated tumors (Figure 4H). These findings reveal the Hippo pathway and pyroptosis as key mediators of SAN's antitumor effects in lung cancer. These findings were further validated by IHC, which revealed that SAN treatment induced YAP nuclear export and degradation, accompanied by significant upregulation of pyroptosis-related proteins, including cleaved caspase-1 and GSDMD (Figures 4J-K, S10A-B). Western blotting, RT-qPCR, and IF analysis consistently confirmed the modulation of Hippo pathway components at both protein and transcriptional levels, in agreement with our sequencing data (Figure S10C, E).

### SAN suppresses lung adenocarcinoma progression by remodeling the tumor microenvironment

Previous studies demonstrated that SAN modulates the tumor microenvironment to exert therapeutic effects in breast cancer and inhibits myeloid-derived suppressor cells in the spleen to influence antitumor immunity 45, 46. These findings prompted us to investigate whether SAN similarly affects the immune microenvironment in LUAD. Single-cell profiling revealed that SAN treatment significantly increased the proportion of CD8^+^ effector T cells within the T cell compartment, rising from 2.08% in control cells to 6.96% in SAN-treated cells (Figure 5A-B). This finding was corroborated by IHC, which revealed enhanced CD8^+^ T cell infiltration in SAN-treated tumors (Figure 5C). GZMB is primarily secreted by cytotoxic T lymphocytes (CTLs), and it functions synergistically with perforin to kill cancer cells, playing a central role in antitumor immunity. Our staining results demonstrated that SAN significantly increased the number of GZMB-positive cells, indicating that SAN promotes the infiltration of CTLs and enhances the antitumor immune response (Figure S10G). Furthermore, cell-cell interaction analysis demonstrated increased communication between CD8^+^ effector T cells and epithelial cells following SAN treatment (Figure 5F), suggesting enhanced immune surveillance. Additionally, the proportion of Th17 cells was higher in the SAN treatment group than in control group, suggesting that SAN might exert antitumor effects through Th17 modulation (Figures 5D, S10F). In addition, the expression of pro-inflammatory factors and some factors that promote the recruitment of immune cells and tissue repair—including *IL1B*, *IL8*, *IL18*, and *IL33,* was significantly increased in lung cancer tissues exposed to SAN (Figure 5E).

Analysis of the distribution preference of single-cell subpopulations revealed that SAN facilitates the enrichment of M1 macrophages (Figures 5G-H). Further analysis of intercellular communication revealed enhanced interactions between specific macrophage subpopulations and epithelial cells (Figure 5I), suggesting that SAN might modulate macrophage-epithelial crosstalk. In contrast to the enhanced immune-epithelial communication, the interaction between tumor-associated fibroblasts and epithelial cells was diminished following SAN treatment, as evidenced by reduced interaction intensity (Figures 5J-K). Collagen plays a critical role in maintaining tumor cell dormancy and facilitating immune evasion, with collagen I (Col1) creating a rigid microenvironment that promotes cancer cell migration, proliferation, and survival through fiber-mediated “tracks” 47, 48. Additionally, α-SMA expression correlates with declining lung function 49, 50. Western blotting revealed that SAN treatment significantly downregulated both Col1 and α-SMA in lung tissues, suggesting that SAN remodels the tumor microenvironment to suppress cancer progression (Figure 5L). Collectively, these findings indicate that SAN suppresses lung cancer by orchestrating the TIME and remodeling tissue architecture.

### Preclinical validation of the antitumor activity SAN in LUAD Organoids

To validate our findings in a clinically relevant model, we next assessed SAN's mechanism and efficacy using patient-derived organoids. Fresh lung cancer specimens were mechanically and enzymatically dissociated into single cells and cultured in a CO_2_ incubator for organoid propagation. Following three passages, the organoids were treated with SAN and subjected to mechanistic and efficacy analyses (Figure 6A). SAN treatment significantly suppressed clonogenicity in human LUAD organoids, consistent with its antitumor efficacy (Figure S11A). Mechanistically, IF analysis revealed that SAN induced the nuclear export of YAP while increasing the cleavage of the pyroptosis markers caspase-1 and GSDMD (Figures 6B-D, S11B-C). Survival curve statistics indicated that high YAP expression was not significantly associated with a lower survival rate in patients with LUAD (Figure 6E). In database analysis, we discovered a negative correlation between YAP and GSDMD expression in patients with LUAD (Figure 6F). Recent studies have revealed that YAP can serve as a therapeutic target in lung cancer. Therefore, SAN, as a drug that targets and degrades YAP, holds great promise for the treatment of LUAD 51-53. These findings recapitulate our observations in cell lines and* in vivo*, further validating SAN's mechanism of action.

This study revealed that SAN, a component of traditional Chinese medicine, inhibits LUAD through a novel mechanism involving Hippo-YAP-mediated pyroptosis. SAN binds to LATS1, activating the Hippo pathway and driving YAP degradation, which triggers gasdermin cleavage, membrane pore formation, and pyroptotic cell death (Figure 6G). This mechanism underlies SAN's ability to suppress LUAD cell proliferation, invasion, and migration. Moreover, *in vivo* studies demonstrated that SAN remodels the tumor microenvironment to enhance its antitumor efficacy. These findings offer valuable insights and support the potential clinical application of SAN for LUAD treatment.

## Discussion

SAN has been revealed to exhibit broad-spectrum antitumor activity across multiple cancer types, although the precise mechanisms remain to be fully defined 54. Our findings established that SAN suppresses LUAD by activating the Hippo pathway to trigger pyroptosis, a previously unrecognized mechanism of action for this compound. These findings add a new dimension to the established model of SAN's antitumor effects, demonstrating that it acts through both proliferation inhibition and pyroptosis induction 8, 12, 55. Our research revealed a distinct function of SAN that extends beyond its activating signaling pathway role, involving a unique compound-protein interaction with LATS1. Through docking in MOE, we demonstrated that SAN engages LATS1 through a dual-mode interaction involving electrostatic anchoring at Asp789 and hydrophobic stabilization around its aromatic core, facilitating LATS1 phosphorylation and YAP degradation to induce pyroptosis. Consistent with this mechanism, YAP overexpression partially rescued cells from SAN-induced pyroptosis, confirming the essential role of YAP degradation in this process (Figure 6G). Importantly, our study established that SAN exerts its antitumor effects through dual mechanisms: Hippo pathway-mediated pyroptosis and tumor microenvironment remodeling.

The Hippo signaling pathway, particularly its transcriptional co-activator YAP, plays a critical role in LUAD progression and therapeutic response. YAP governs tissue growth and organ size by regulating stem and progenitor cell proliferation. Consequently, its dysregulation promotes tumorigenesis. In addition to its well-documented function in driving EMT, YAP has also been revealed to modulate apoptotic pathways, highlighting its multifaceted role in cancer biology 56-60. This study uncovers a novel mechanism by which SAN regulates pyroptosis through YAP. For the first time, we demonstrated that YAP directly controls pyroptosis in LUAD cells, extending previous reports that implicated the Hippo pathway in this process 42, 43. YAP is a clinically actionable target in various cancers 58, 61. Our findings highlight SAN as a novel modulator of YAP activity, suggesting its potential as a therapeutic candidate to improve outcomes in LUAD.

Pyroptosis is a type of programmed cell death mediated by inflammasomes and executed by gasdermins. This process is characterized by membrane perforation, intracellular content release, and a robust inflammatory response. Emerging evidence links the Hippo pathway to pyroptosis regulation. For instance, MST1 deletion in intestinal epithelial cells promotes YAP nuclear translocation, enhancing YAP/p73-mediated transcription of caspase-1 and inducing pyroptosis 19. Conversely, MST1/2 deletion directly triggers macrophage pyroptosis, whereas gut microbiota-mediated Hippo regulation inhibits caspase-3-GSDMD-dependent pyroptosis via BCL2 upregulation 42, 62. These findings establish the Hippo pathway as a key regulator of pyroptosis. Building on this, we demonstrated that SAN, by promoting LATS1 phosphorylation, directly modulates YAP activity to control pyroptosis in LUAD cells, revealing a novel mechanism of Hippo-mediated pyroptosis regulation.

Our study has provided compelling evidence of the role of SAN in remodeling the TIME during LUAD treatment. Prior research offered limited insights into the mechanism by which SAN modulates immune cells *in vivo*, including its effects on T cells, macrophages, and fibroblasts, as well as their interactions with epithelial cells. To address this gap, we demonstrated for the first time that SAN inhibits epithelial cell proliferation and induces pyroptosis via Hippo pathway regulation, and it also enhances antitumor immunity by increasing the proportions of CD8^+^ T cells and macrophages. Furthermore, SAN strengthens intercellular communication among T cells, macrophages, and epithelial cells while reducing the abundance of tumor-associated fibroblast interactions. Accumulating evidence indicates that pyroptosis in tumor cells is closely linked to immune microenvironment remodeling 63, 64. Given that pyroptosis produces substantial pro-inflammatory cytokines, including IL-1β and IL-18, we propose that the resultant cytokine alterations initiate a cascade of events that modulate chemokines and other critical immune regulators in the tumor microenvironment. Ultimately, this cascade contributes to microenvironment remodeling and suppresses tumor progression. Conversely, the remodeled immune microenvironment might in turn trigger pyroptosis via the generation of pro-inflammatory cytokines, thereby further inhibiting lung cancer progression. These findings indicate our future direction, in which we will further assess whether combining SAN with immune checkpoint inhibitors can yield additional therapeutic benefits for LUAD.

Our finding of a positive correlation between p-YAP and GSDMD-N expression suggests their interaction in pyroptosis. Given YAP's established roles in proliferation and apoptosis and GSDMD's function in pyroptosis 65, 66, we hypothesize that YAP regulates GSDMD cleavage in LUAD through phosphorylation-dependent of YAP. Mechanistically, YAP can either enhance caspase-1 transcription via nuclear co-factors or, upon cytoplasmic retention, promote inflammatory signaling through phase separation. Future work should delineate these possibilities to fully understand the crosstalk between Hippo signaling and pyroptosis. Our research demonstrated that SAN inhibits LUAD through two mechanisms: Hippo-mediated pyroptosis and immune microenvironment remodeling. These findings establish SAN as a promising therapeutic candidate and open several avenues for future investigation. First, the precise molecular interaction between SAN and LATS1, including the specific phosphorylation sites involved, remains to be determined. Second, whether p-YAP regulates GSDMD cleavage in a site-specific manner warrants further exploration. Third, the potential impact of SAN on immune microenvironments in other lymphoid organs, such as the spleen and lymph nodes, merits investigation. Addressing these questions will provide a more comprehensive understanding of SAN's antitumor mechanism.

In conclusion, our work revealed a novel mechanism by which the Chinese herbal monomer SAN exerts its tumor-suppressive effects, as verified across multiple models. Mechanistically, SAN binds to LATS1, promoting its phosphorylation and subsequent YAP nuclear export and degradation, thereby inducing pyroptosis. Meanwhile, SAN remodels the tumor microenvironment to facilitate the cold-to-hot tumor transition, ultimately enhancing the antitumor immune response. These mechanisms have been validated across multiple experimental platforms, ranging from cell lines and mouse models to patient-derived organoids. A limitation of this study was the lack of clinical efficacy evaluation; however, our findings provide a strong rationale for future clinical trials of SAN in LUAD.

## Supplementary Material

Supplementary figures and methods.

## Figures and Tables

**Figure 1:**
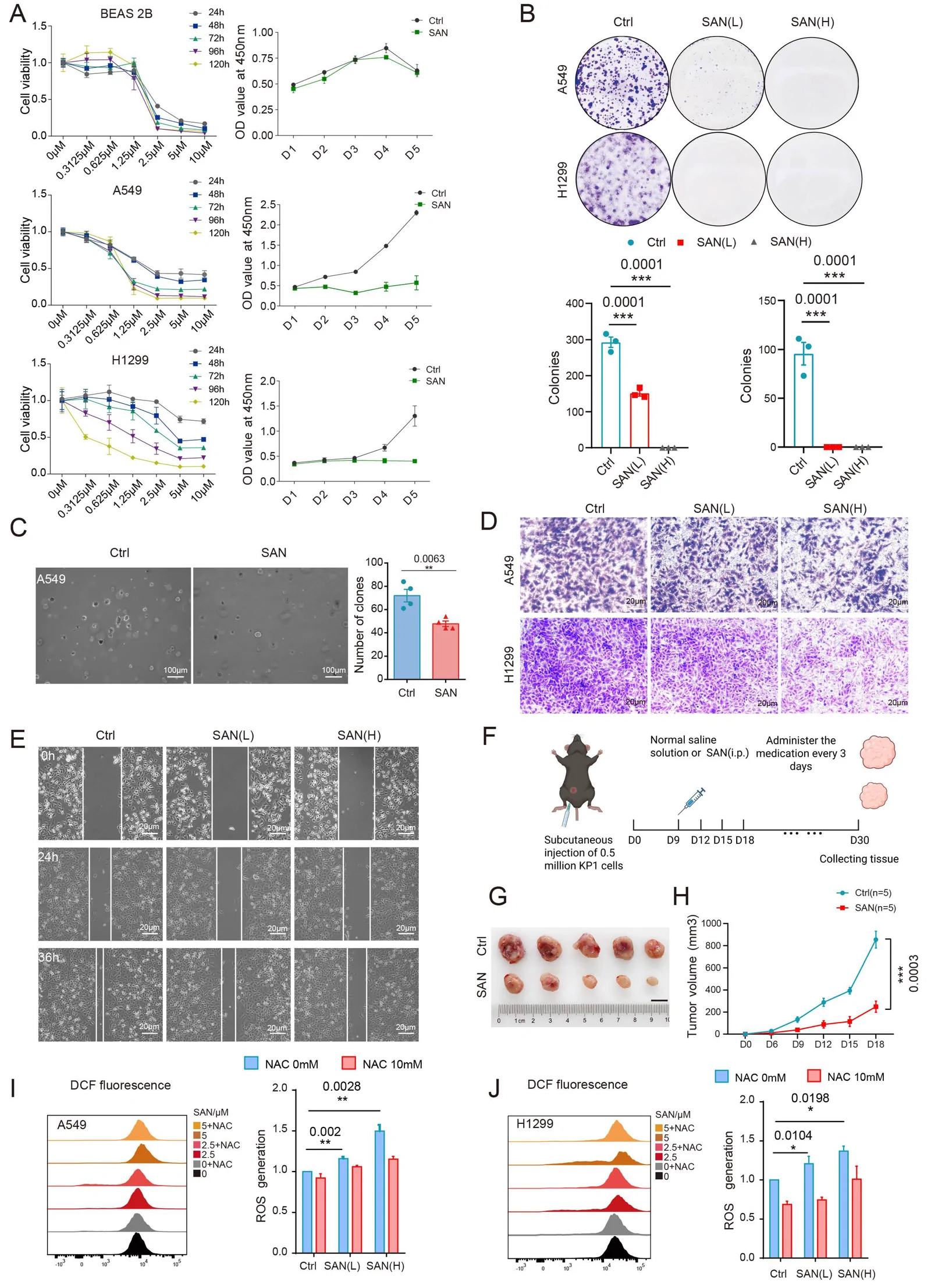
** Sanguinarine (SAN) inhibits the proliferation, clonal formation, and migration of lung cancer cells and promotes reactive oxygen species (ROS) production.** (A) Proliferation of BEAS-2B normal epithelial cells and A549 and H1299 lung adenocarcinoma cells. (B) Analysis of the clonogenic ability of A549 and H1299 cells. (C) The soft agar colony formation assay was performed to assess the effect of SAN (2.5 μM) or vehicle (DMSO) on colony formation. Scale bars, 100 μm. (D) Transwell migration assay was performed to assess the effect of SAN (1.5 or 3 μM) or vehicle (DMSO) on cell migration. Scale bars, 20 μm. (E) The cell scratch assay was performed to assess the effect of SAN (1.5 or 3 μM) or vehicle (DMSO) on the A549 cell migration. Scale bars, 20 μm. (F) Schematic representation of the subcutaneous tumor formation assay performed using SAN. (G and H) Subcutaneous tumors were treated with SAN, and tumor size was monitored (n = 5). (I and J) After treating A549 and H1299 cells with SAN, ROS production and N-acetylcysteine (NAC) rescue effects were assessed. Data in (B) were analyzed with Brown-Forsythe and Welch analysis of variance (ANOVA) test with Dunnett's multiple comparisons test. *, *P* < 0.05, **, *P* < 0.01.

**Figure 2:**
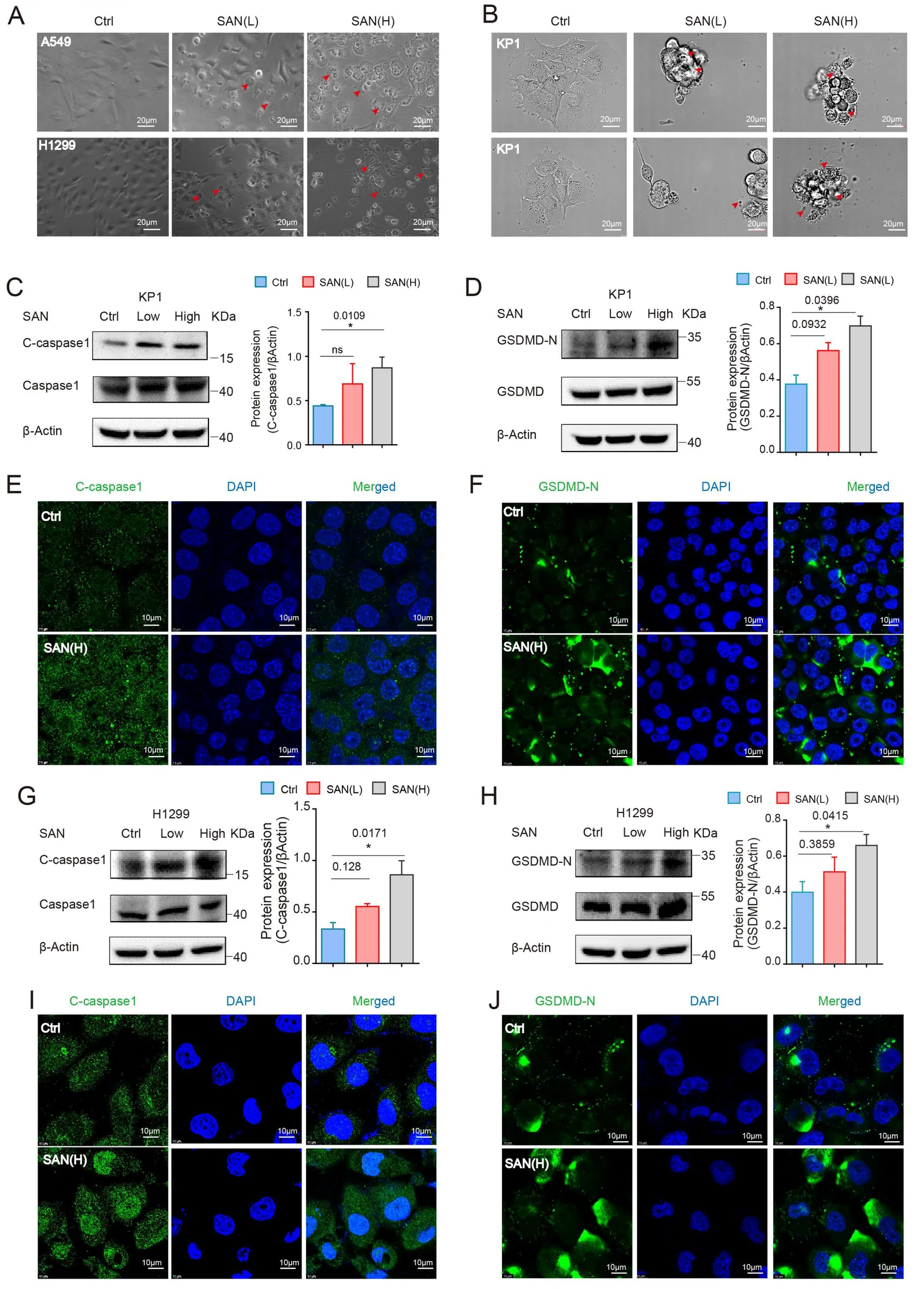
** SAN induces pyroptosis in LUAD cells.** (A and B) Brightfield microscopy reveals bubble-like structures indicative of pyroptosis formed in A549, H1299, and KP1 cells treated with SAN. Scale bars, 20 μm. (C and D) Western blotting of KP1 cell lysates.** (**E and F) Immunofluorescence staining analysis of KP1 cells treated with SAN (3 μM) or vehicle (DMSO). Scale bars, 10 μm. (G and H) Western blotting of H1299 cell lysates.** (**I and J) Immunofluorescence staining analysis of H1299 cells treated with SAN (3 μM) or vehicle (DMSO). Scale bars, 10 μm. Data in (C), (D), (G) and (H) were analyzed with Brown-Forsythe and Welch analysis of variance (ANOVA) test with Dunnett's multiple comparisons test. *, *P* < 0.05, **, *P* < 0.01.

**Figure 3:**
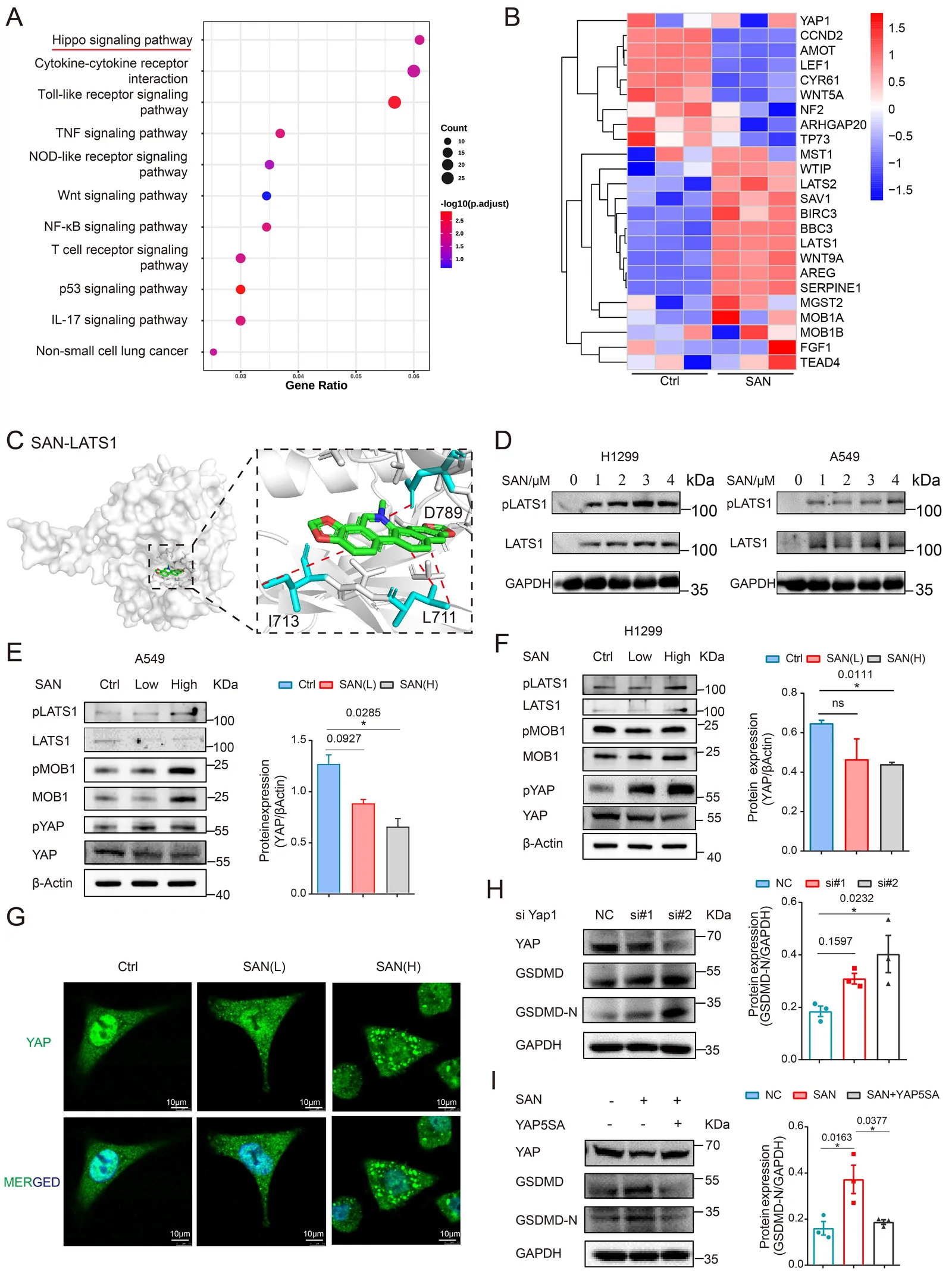
** SAN induces pyroptosis mediated by the Hippo pathway. (**A) Differential gene expression of Hippo signaling pathway components was assessed by RNA-seq using A549 cells treated with SAN (5 μM) or vehicle (DMSO). (B) Transcriptome-based differential expression analysis was conducted to compare the expression profiles of genes involved in the Hippo signaling pathway between SAN-treated and untreated A549 cells. (C) Molecular docking reveals the specific interaction between SAN and large tumor suppressor 1 (LATS1).** (**D) Western blotting of H1299 and A549 cells treated with various concentrations of SAN. (E and F) Western blotting of H1299 and A549 cells treated with SAN (1.5 or 3 μM) or vehicle (DMSO).** (**G) Immunofluorescence staining analysis of A549 cells treated with SAN (1.5 or 3 μM) or vehicle (DMSO). Scale bars, 10 μm. (H) Western blotting of KP1 cell lysate after the knockdown of *YAP* by siRNA.** (**I) Western blotting of H1299 cell lysate after treatment with SAN (3 μM, 4h) or overexpression of YAP. Data in (E), (F), (H) and (I) were analyzed with Brown-Forsythe and Welch analysis of variance (ANOVA) test with Dunnett's multiple comparisons test. *, *P* < 0.05, **, *P* < 0.01.

**Figure 4:**
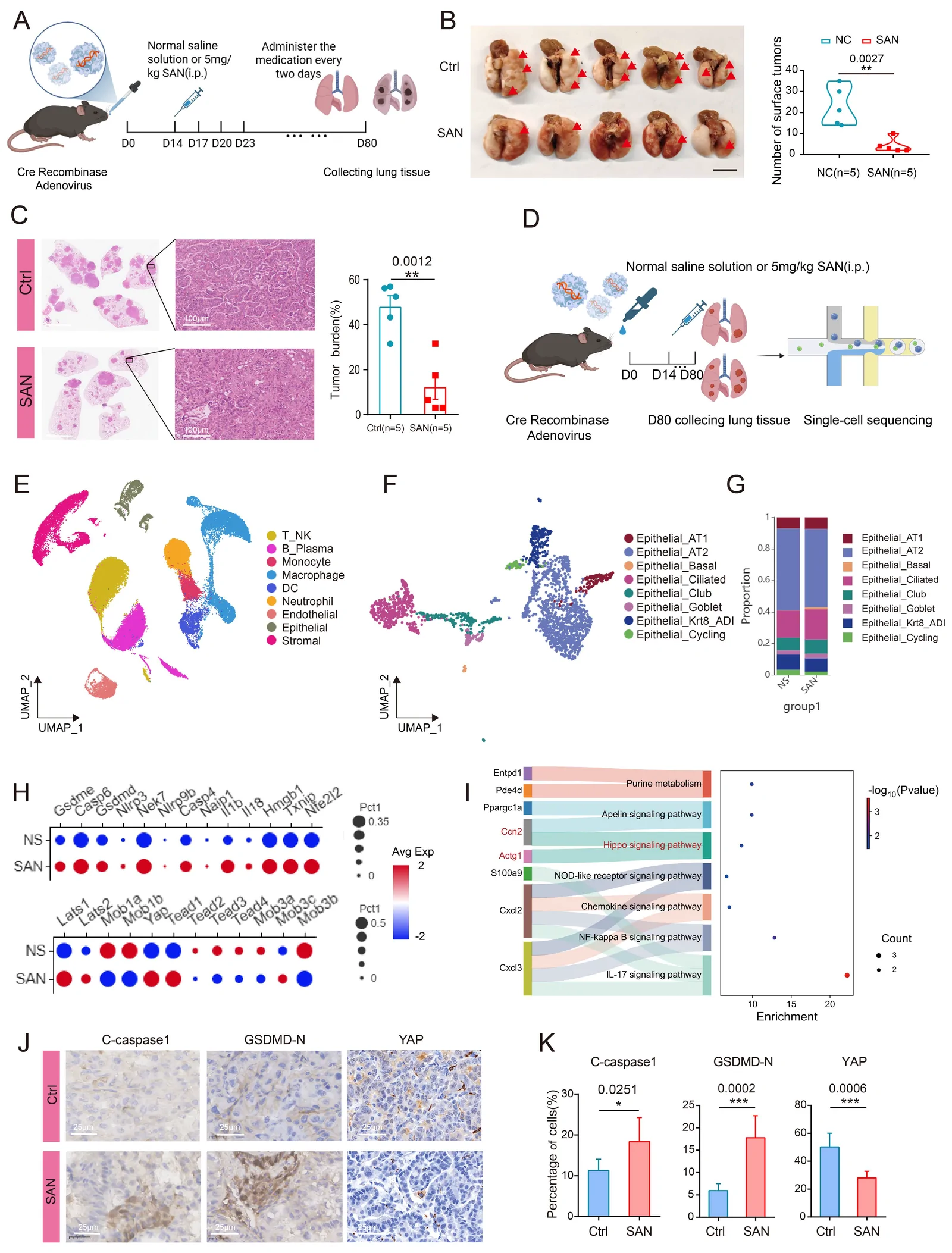
** SAN suppresses tumor progression *in vivo* via Hippo-mediated epithelial cell pyroptosis. (**A) Schematic representation of the *Kras^ LSL-G12D/+^; Trp53^fl/fl^* mouse model of primary lung cancer treated with SAN. (B) Lung tissue with tumors along with the statistical analysis of the surface tumor (n = 5).** (**C) HE staining of lung cancer tumor growth in mice in the SAN and control group (n = 5). Scale bars, 100 μm. (D) Schematic representation of the single-cell sequencing process. (E) The distribution of cell groups based on the results of single-cell sequencing. (F and G) Classification and proportion of epithelial cell subgroups. (H) Bubble chart presenting the expression changes of key genes in the Hippo pathway and genes related to cell pyroptosis.** (**I) Differential expression in genes involved in the Hippo signaling pathway was identified by single-cell sequencing analysis. (J and K) Immunohistochemistry of cleaved caspase-1, gasdermin D N-terminal fragment (GSDMD-N), and YAP in mouse lung cancer tissues treated with SAN or normal saline. Scale bars, 25 μm. Statistical significance was determined using Student's* t-*test. *, *P* < 0.05; n.s., not significant.

**Figure 5:**
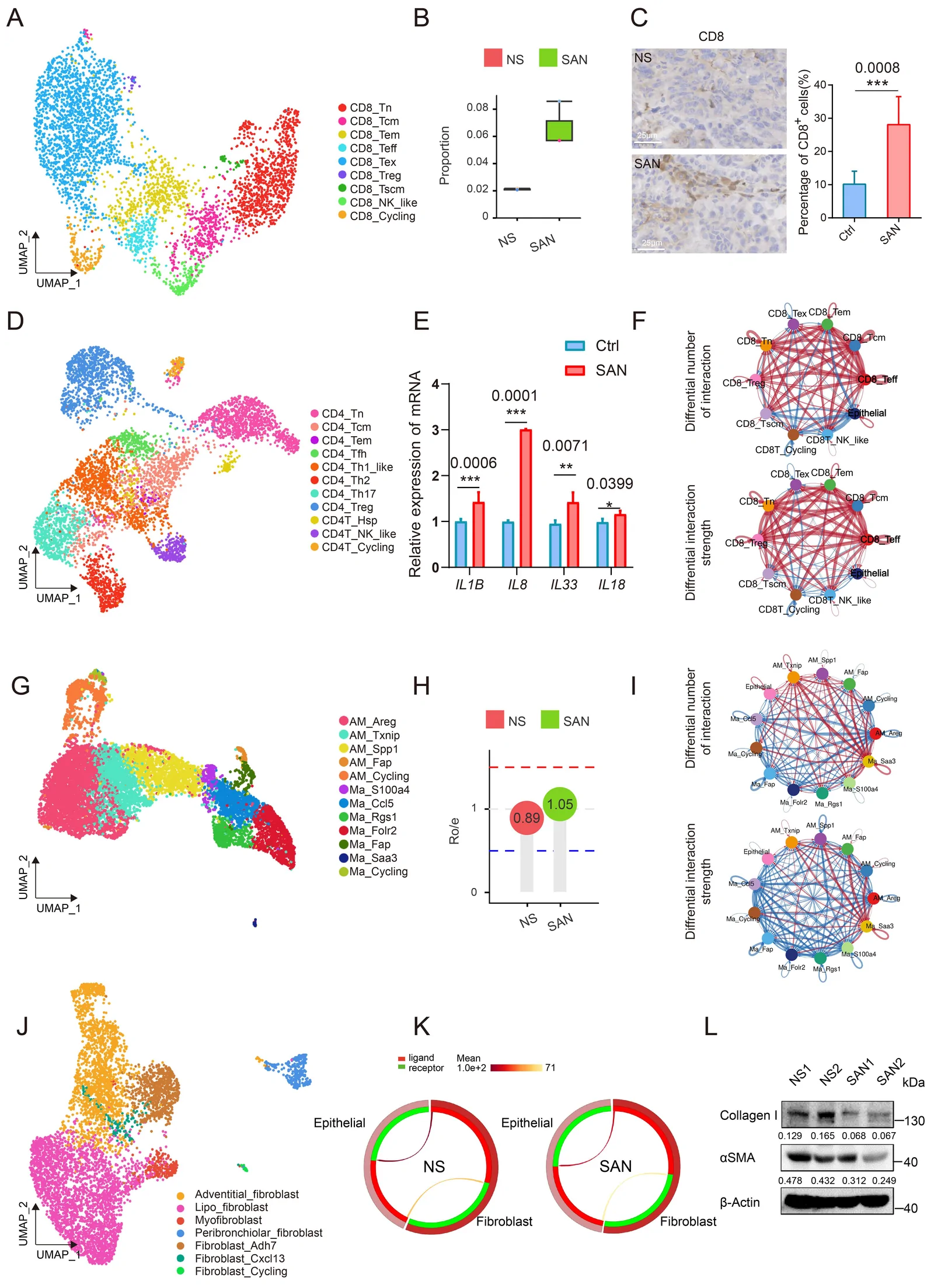
** SAN regulates the tumor immune microenvironment *in vivo* to resist tumor growth. (**A and B) Classification of CD8^+^ T subsets and changes in the proportion of CD8^+^ effector T cells. (C) Immunohistochemistry of CD8 in mouse lung cancer tissues treated with SAN or normal saline. Scale bars, 25 μm. Statistical significance was determined using Student's* t-*test. *, *P* < 0.05; n.s., not significant.** (**D) CD4^+^ T cell subset classification and changes in cell proportions.** (**E) Detection of cytokines released during pyroptosis with potential effects on the immune microenvironment in mouse lung cancer tissues treated with SAN or normal saline. (F) Quantitative and qualitative changes in the interaction between CD8^+^ T cells and epithelial cells. (G and H) Classification of macrophage subpopulations and tissue preference of M1 macrophages.** (**I) Quantitative and qualitative changes in the interaction between macrophages and epithelial cells. J) Classification of fibroblast subpopulations. (K) Changes in the interaction between fibroblasts and epithelial cells.** (**L) Western blotting analysis of mouse lung cancer tissue lysate.

**Figure 6:**
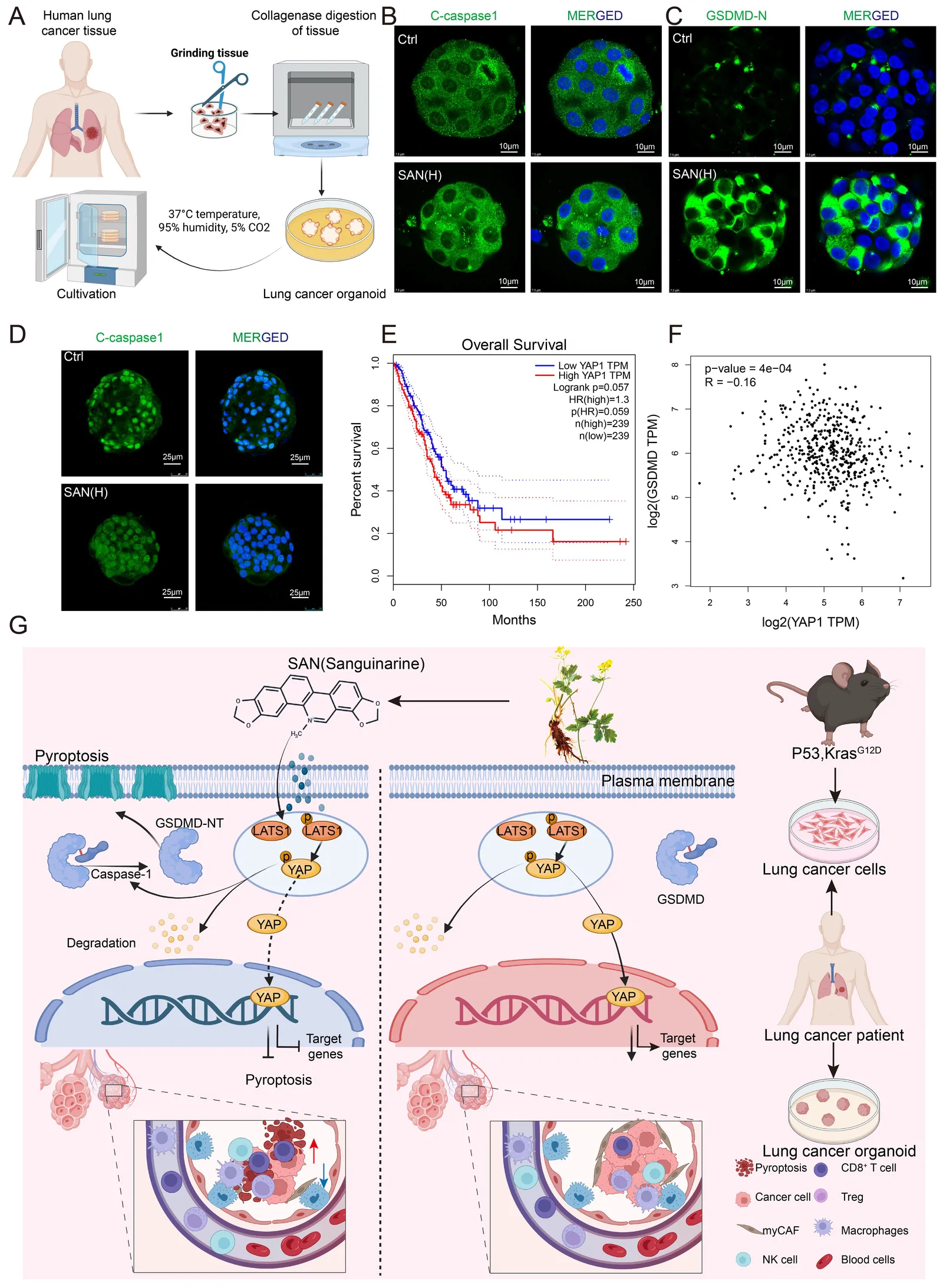
** SAN activates the Hippo pathway in human lung cancer organoids and promotes cell pyroptosis.** (A) Schematic representation of human lung cancer organoid cultivation. (B and C) Immunofluorescence staining of cleaved caspase1 and gasdermin D N-terminal fragment (GSDMD-N) in human-derived lung cancer organoids treated with SAN (3 μM) or vehicle (DMSO). Scale bars, 10 μm. (D) Immunofluorescence staining analysis of YAP in human-derived lung cancer organoids treated with SAN (3 μM) or vehicle (DMSO). Scale bars, 25 μm. (E) Correlation analysis of YAP expression and survival in patients with lung adenocarcinoma. (F) The relationship between YAP and GSDMD expression in patients with lung adenocarcinoma. (G) Mechanism diagram.

## Abbreviations

LUAD: Lung adenocarcinoma
LATS1: Large Tumor Suppressor 1
SAN: Sanguinarine
CRC: colorectal cancer
NSCLC: non-small cell lung cancer
TKI: tyrosine kinase inhibitor
NOX3: NADPH oxidase 3
DAMPs: damage-associated molecular patterns
CuB: cucurbitacin B
TIME: tumor immune microenvironment
MOE: Molecular Operating Environment
CTLs: cytotoxic T lymphocytes
Col1: collagen I
