# Supplementary material for: Sanguinarine Induces Pyroptosis via the Hippo Signaling Pathway and Remodels the Tumor Immune Microenvironment in Lung Adenocarcinoma
Source: Int J Biol Sci. 2026 Oct 2;22(15):8668–84. doi: 10.7150/ijbs.139061 (PMC13686061; doi:10.7150/ijbs.139061)
Supplement: Supplementary file 1 [file ijbsv22p8668s1.pdf]

Sanguinarine Induces Pyroptosis *via* the Hippo Signaling Pathway and Remodels the Tumor Immune Microenvironment in Lung Adenocarcinoma

Jia Yang[1,#], Lanfang Li[2,#], Yunjian Pan[3] , Wulei Hou[2], Xiaoyan Wang[4], Zhengda Li[2], Wenjie Wang[6], Yihua Sun[3], Ping Xu[1], Lifang Wang[1], Ying Lu[7], Zhongqi Wang[1], Zhenhui Lu[5,*], Zuoyun Wang[2,*]

[1]Department of Oncology, Longhua Hospital Shanghai University of Traditional Chinese Medicine, Shanghai 200032, China

[2]Department of Anatomy, Histology and Embryology, School of Basic Medical Sciences, Shanghai Pudong Hospital, Fudan University, Shanghai 200032, China

[3]Fudan University Shanghai Cancer Center, Shanghai 200032, China

4Huadong Hospital Affiliated to Fudan University, Shanghai 200040, China

[5]Institute of Respiratory Diseases, Longhua Hospital Shanghai University of Traditional Chinese Medicine, Shanghai 200032, China

[6]Bayinguoleng Mongolian Autonomous Prefecture Hygiene School, Xinjiang Province 841000, China

[7]Department of Biochemistry and Molecular Biology, School of Basic Medical Sciences, Fudan University, Shanghai 200032, China

[#] Equally Contributing Authors

[*] Correspondence authors:

Dr. Zuoyun Wang at Department of Anatomy, Histology and Embryology, School of Basic Medical Sciences, Shanghai Pudong Hospital, Fudan University, Shanghai 200032, China. Email: wangzuoyun@fudan.edu.cn

Dr. Zhenhui Lu at Institute of Respiratory Diseases, Longhua Hospital Shanghai University of Traditional Chinese Medicine, Shanghai 200032, China. Email: Dr_luzh@shutcm.edu.cn

Supplementary figure1-11

Supplementary Figure 1

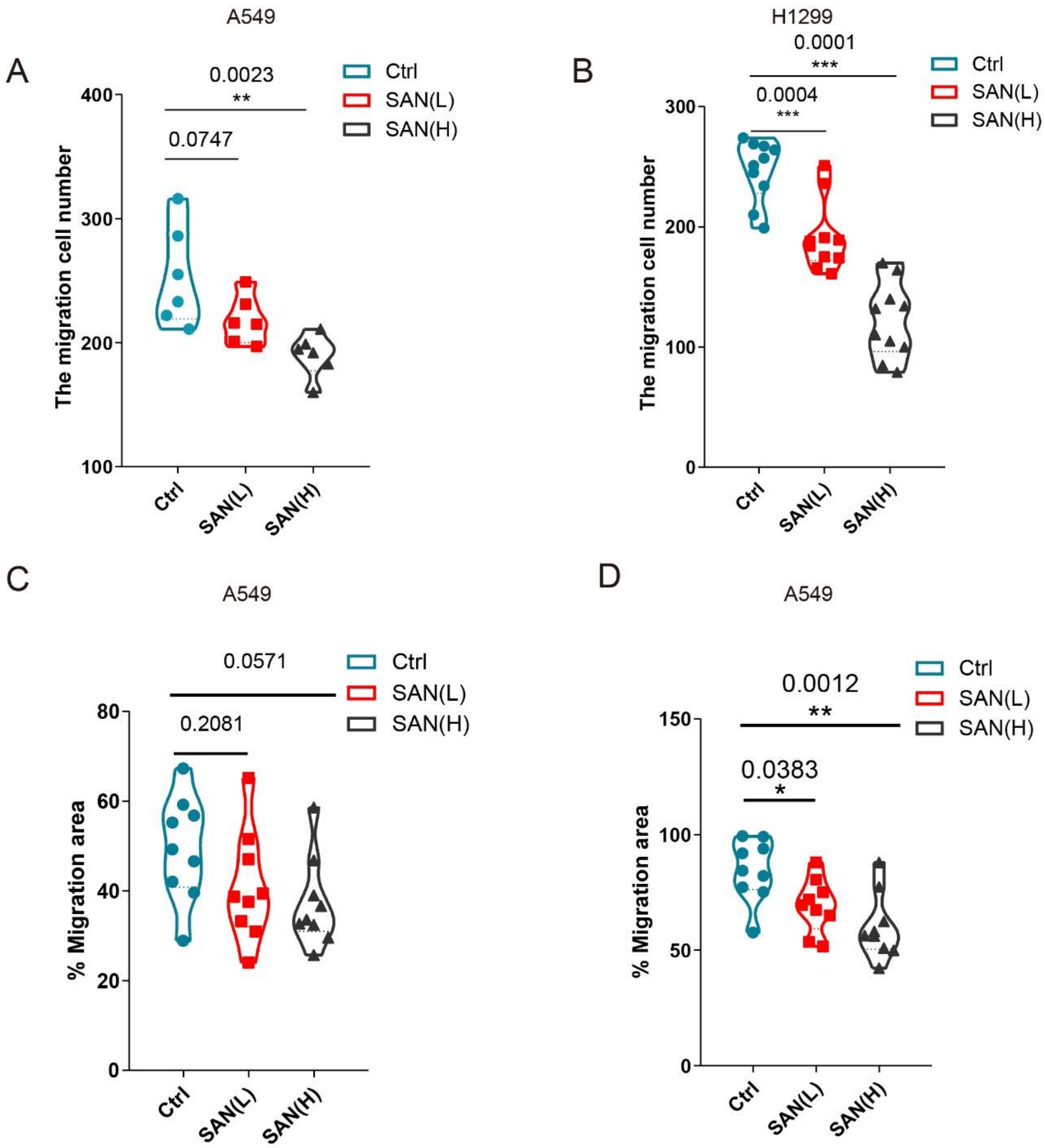

Supplementary Figure 1. Statistical analysis of the migration scratch assays. (A and B) Statistical analysis of the migration assay with A549 and H1299 cells. (C and D) Statistical analysis of the scratch experiment using A549 cells after 24 and 36h. Data in (A), (B), (C) and (D) were analyzed with Brown-Forsythe and Welch analysis of variance (ANOVA) test with Dunnett's multiple comparisons test. *, $P < 0.05$, **, $P < 0.01$; n.s., not significant.

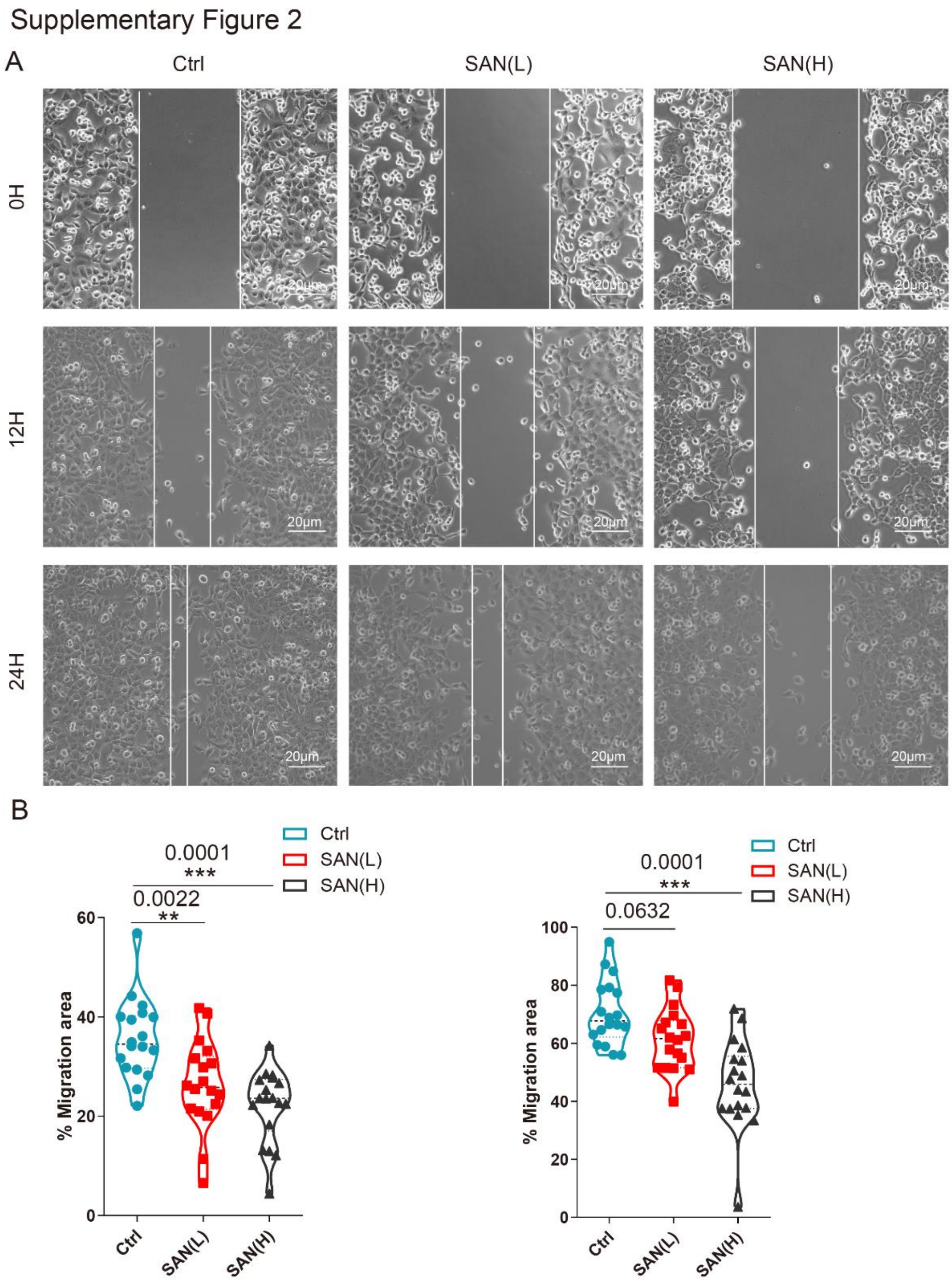

Supplementary Figure 2. Statistical analysis of the scratch experiment. (A and B) Statistical analysis of the scratch experiment using H1299 cells after 12 and 24h. Scale bars, 100 μm. Data in (B) and (C) were analyzed with Brown-Forsythe and Welch analysis of variance (ANOVA) test with Dunnett's multiple comparisons test. *, $P < 0.05$, **, $P < 0.01$; n.s., not significant.

Supplementary Figure 3

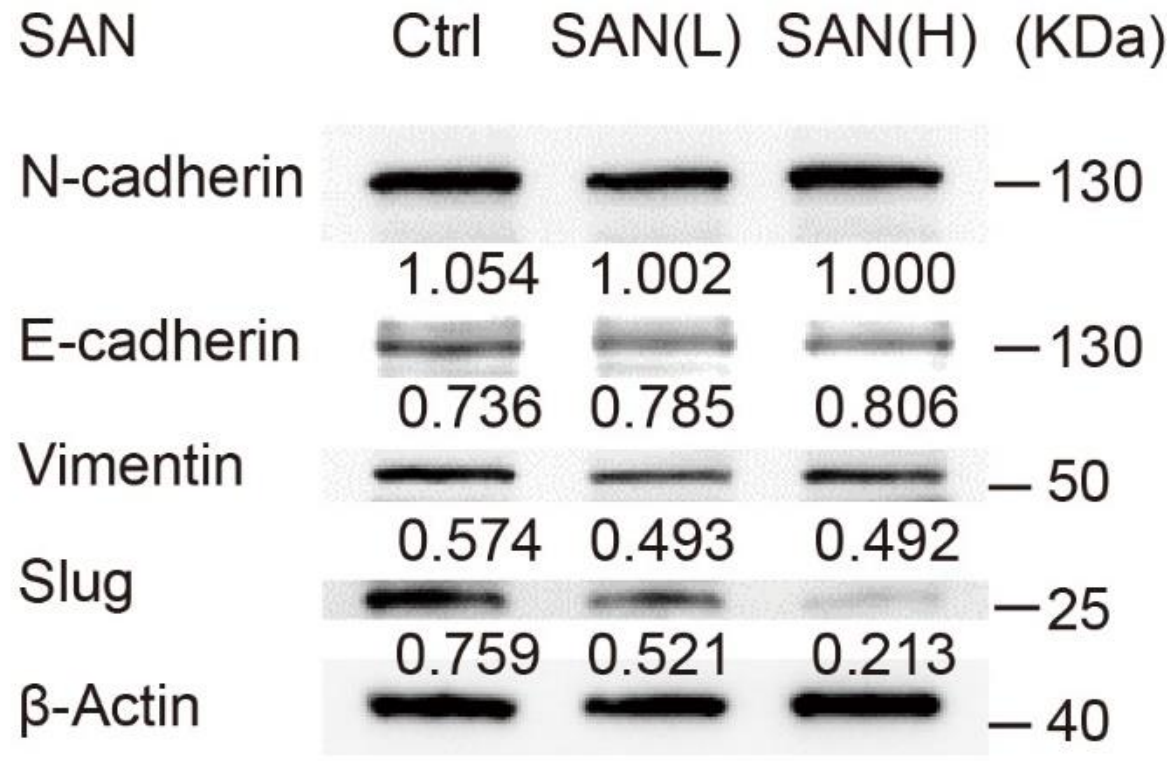

Supplementary Figure 3. Western blotting of lung cancer cell lysate after treatment with sanguinarine (SAN) in A549 cells.

Supplementary Figure 4

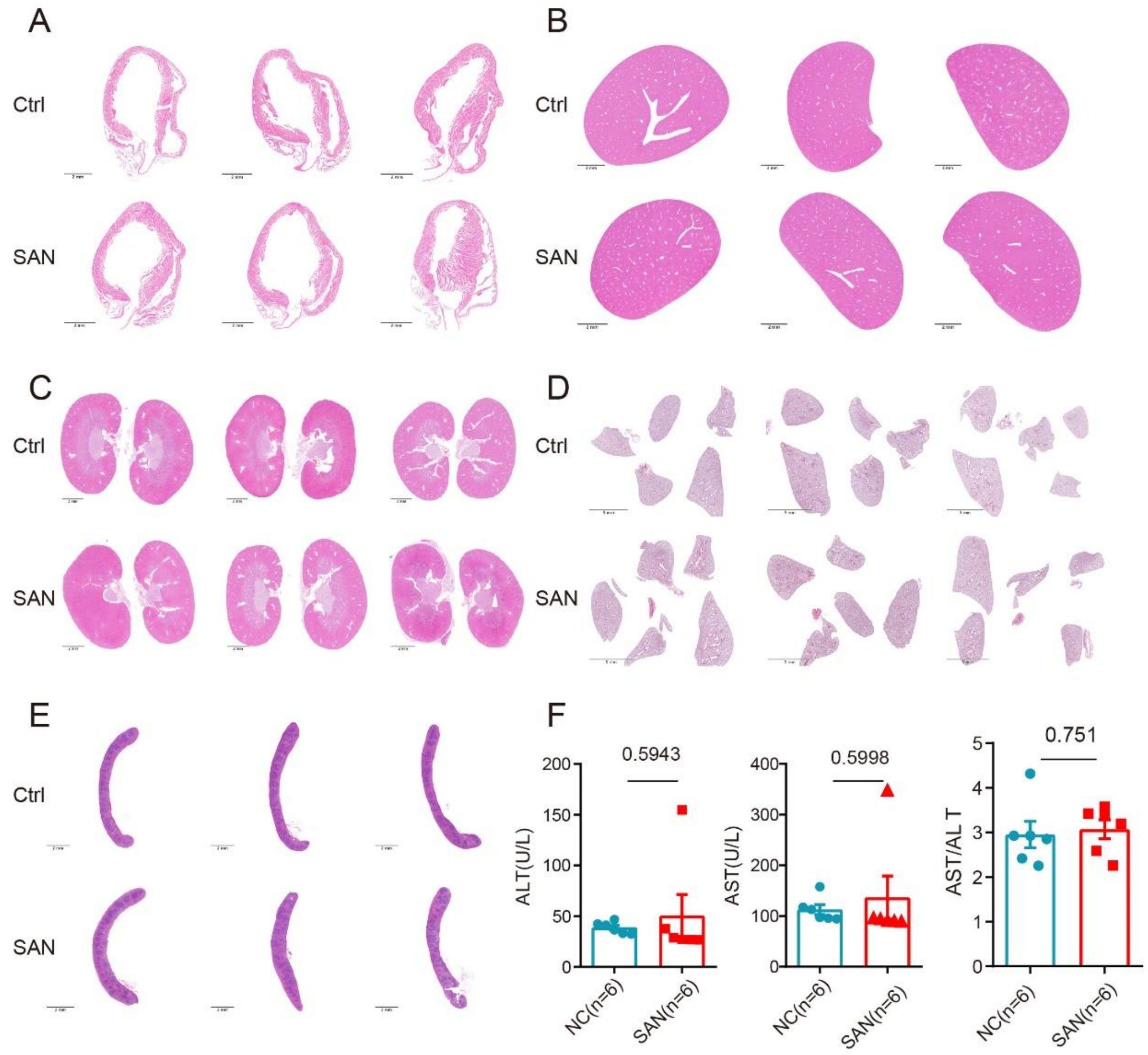

Supplementary Figure 4. Sanguinarine (SAN) safety assessment.

(A - C) The histological architecture of the heart, liver, and kidneys was evaluated by H&E staining in mice treated with SAN compared to those treated with normal saline. Scale bars, 2mm. **(**D) The histological architecture of the lungs was evaluated by H&E staining in mice treated with SAN compared to those treated with normal saline. Scale bars, 5mm. **(**E) The histological architecture of the spleen was evaluated by H&E staining in mice treated with SAN compared to those treated with normal saline. Scale bars, 2mm. (F) Serum levels of alanine aminotransferase (ALT) and aspartate aminotransferase (AST) were assessed in mice treated with SAN compared to those treated with normal saline. Statistical significance was determined using Student's *t*-test. *, $P < 0.05$; n.s., not significant.

Supplementary Figure 5

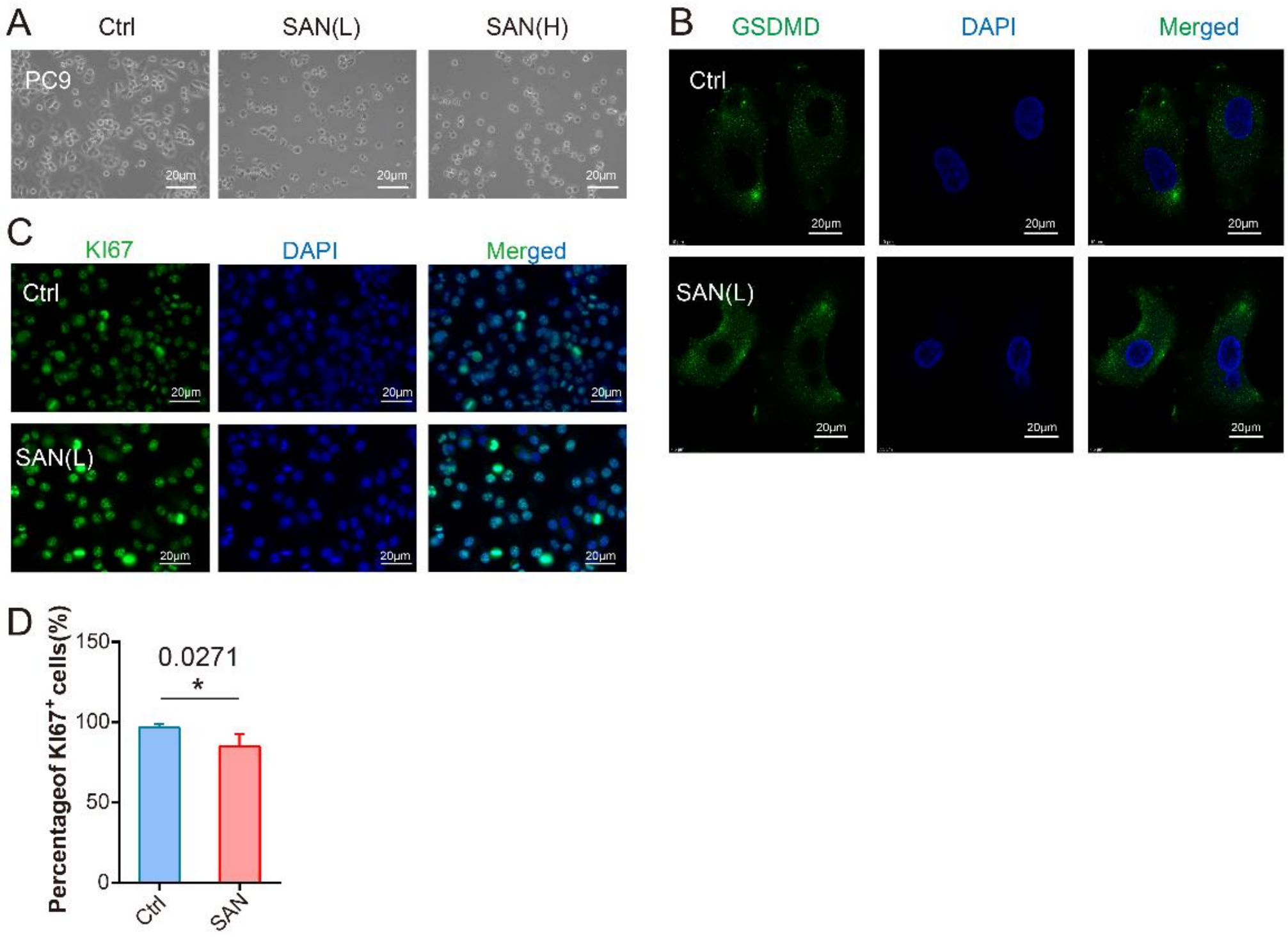

Supplementary Figure 5. Sanguinarine (SAN) promotes pyroptosis of lung cancer cells. (A) Brightfield microscopy results illustrate that PC9 cells treated with SAN formed bubble-like structures. Scale bars, 20 μm. (B) Immunofluorescence staining of gasdermin D (GSDMD) in A549 treated with SAN (1.5 μM) or vehicle (DMSO). Scale bars, 20 μm. (C and D) Immunofluorescence staining of Ki-67 in H1437 cells treated with SAN (1.5 μM) or vehicle (DMSO). Scale bars, 20 μm. Statistical significance was determined using Student's *t*-test. *, $P < 0.05$; n.s., not significant.

Supplementary Figure 6

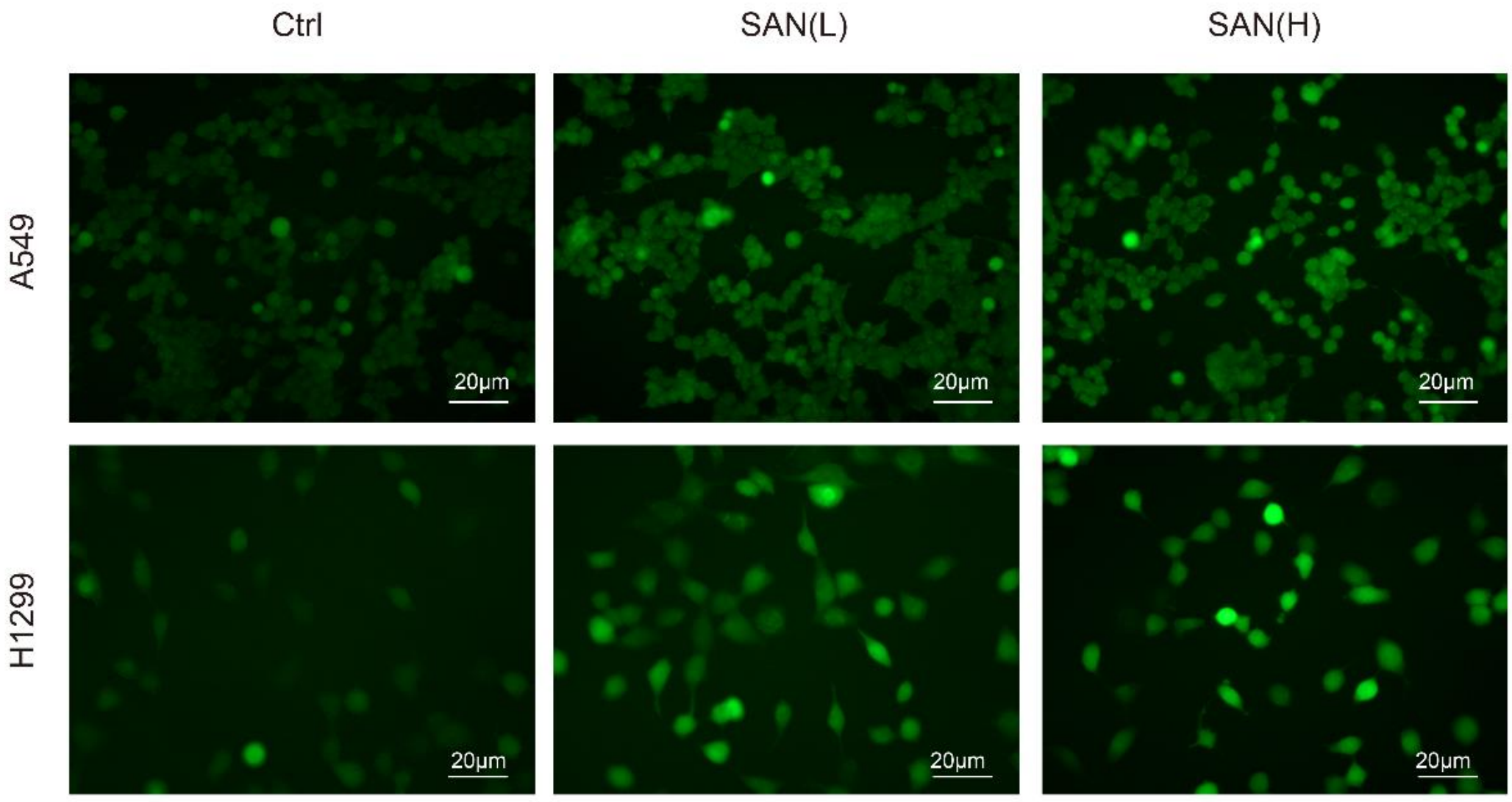

Supplementary Figure 6. Detection of reactive oxygen species production was promoted by sanguinarine (SAN) treatment in lung cancer cells. Scale bars, 20 μm.

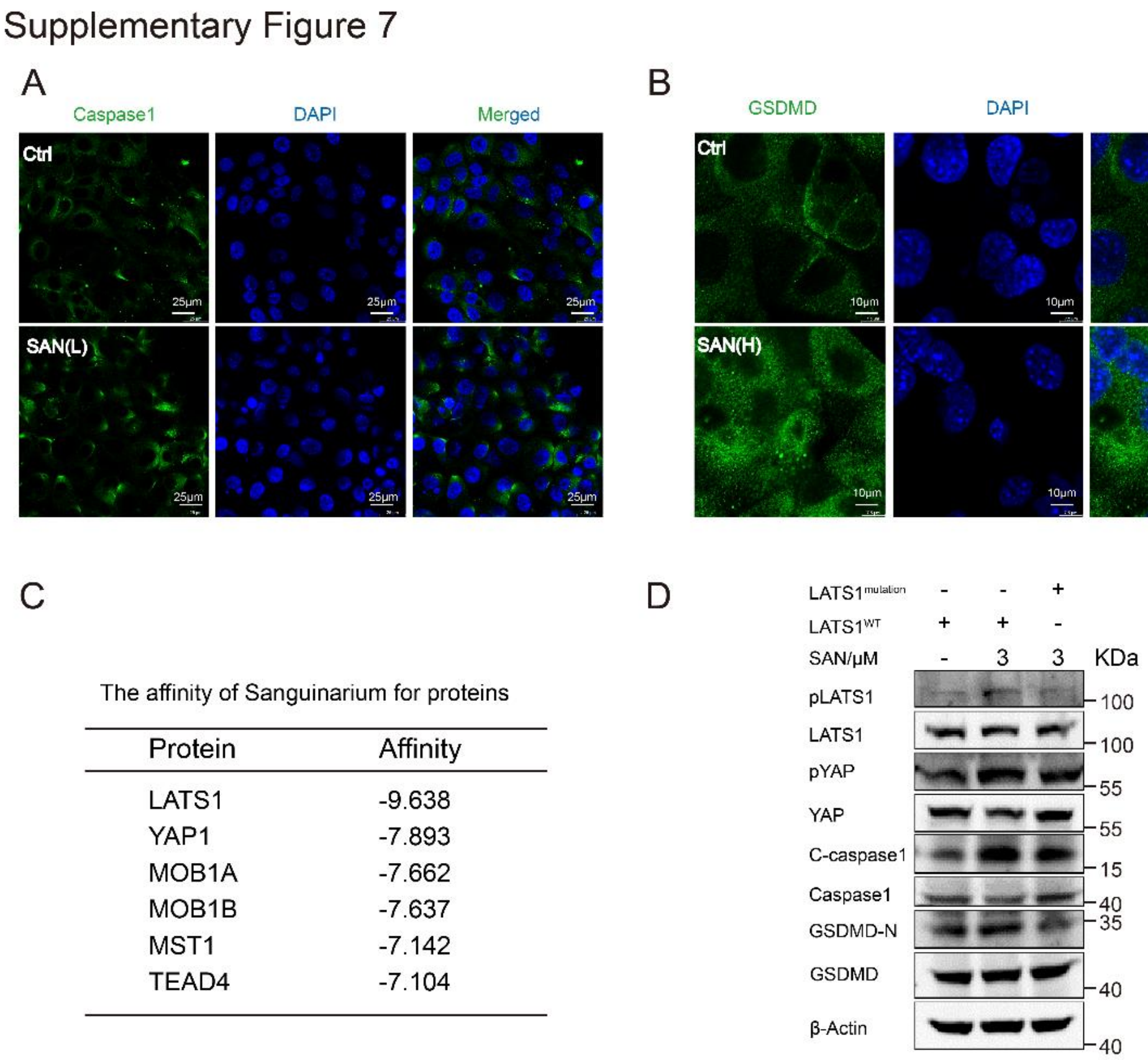

Supplementary Figure 7. Sanguinarine (SAN) inhibits the expression of pyroptosis-related proteins in lung cancer cells. (A) Immunofluorescence staining analysis of Caspase1 in KP1 cells treated with SAN or vehicle (DMSO). Scale bars, 25 μm. **(**B) Immunofluorescence staining analysis of gasdermin D (GSDMD) in KP1 cells treated with SAN or vehicle (DMSO). Scale bars, 10 μm. (C) The ranking of protein interaction intensities between SAN and Hippo signaling components. (D) Western blotting was used to detect the changes in the expression levels of Hippo pathway and pyroptosis-related proteins in the H1299 cell lysates after respectively transfected with $LATS1^{WT}$, $LATS1^{mutation}$, and treated with SAN.

Supplementary Figure 8

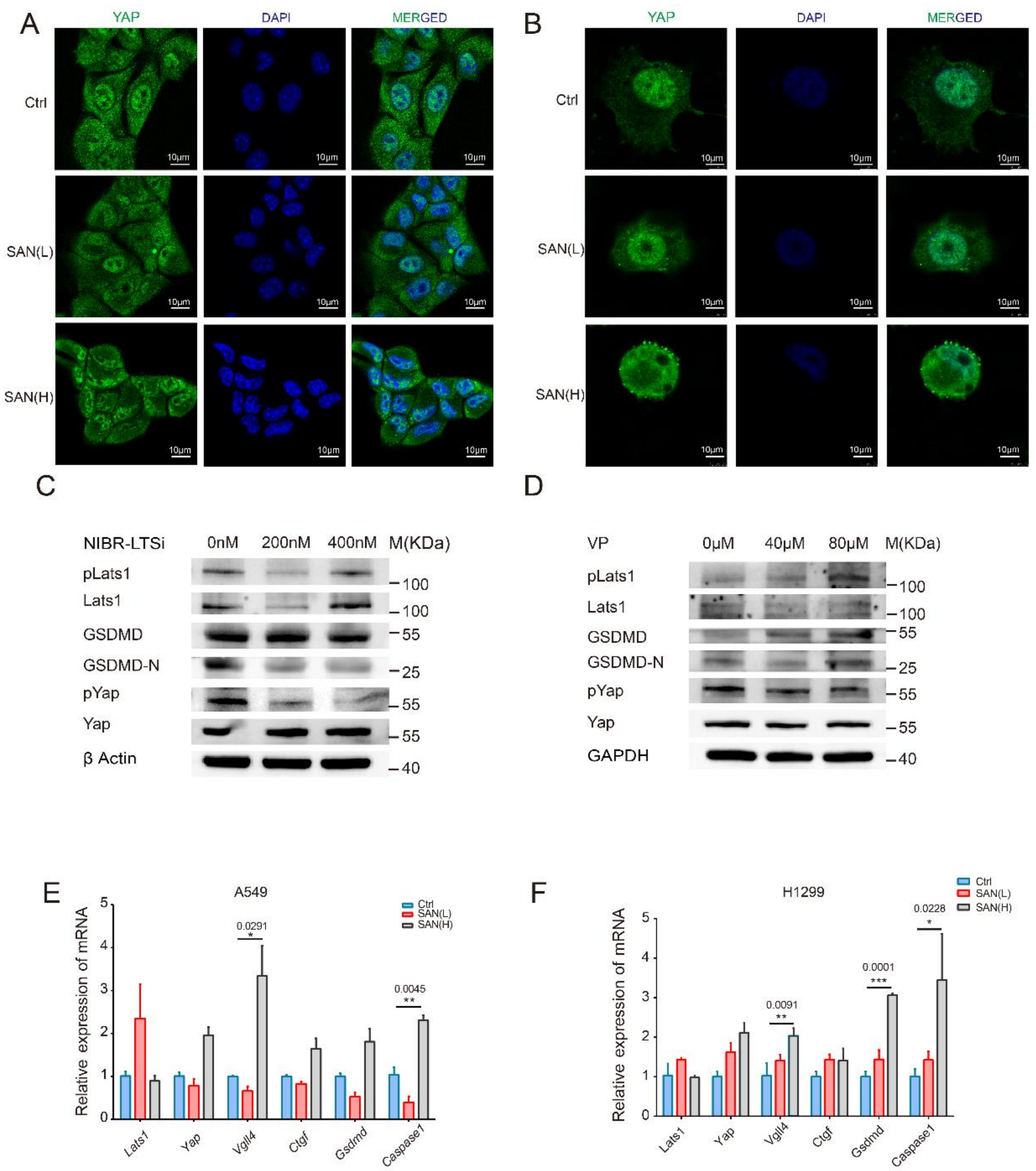

Supplementary Figure 8. Sanguinarine (SAN) induces cell pyroptosis through the Hippo pathway in lung cancer cells. (A and B) Immunofluorescence staining analysis of YAP in KP1 cells and H1299 cells treated with SAN (1.5 or 3 μM) or vehicle (DMSO). Scale bars, 10 μm. (C and D) Western blotting of A549 cells lysate after treatment with NIBR-LTSi or verteporfin. (E and F) RT-qPCR of A549 and H1299 cells after treatment with SAN. Statistical significance was determined using Student's *t*-test. *, $P < 0.05$; n.s., not significant.

Supplementary Figure 9

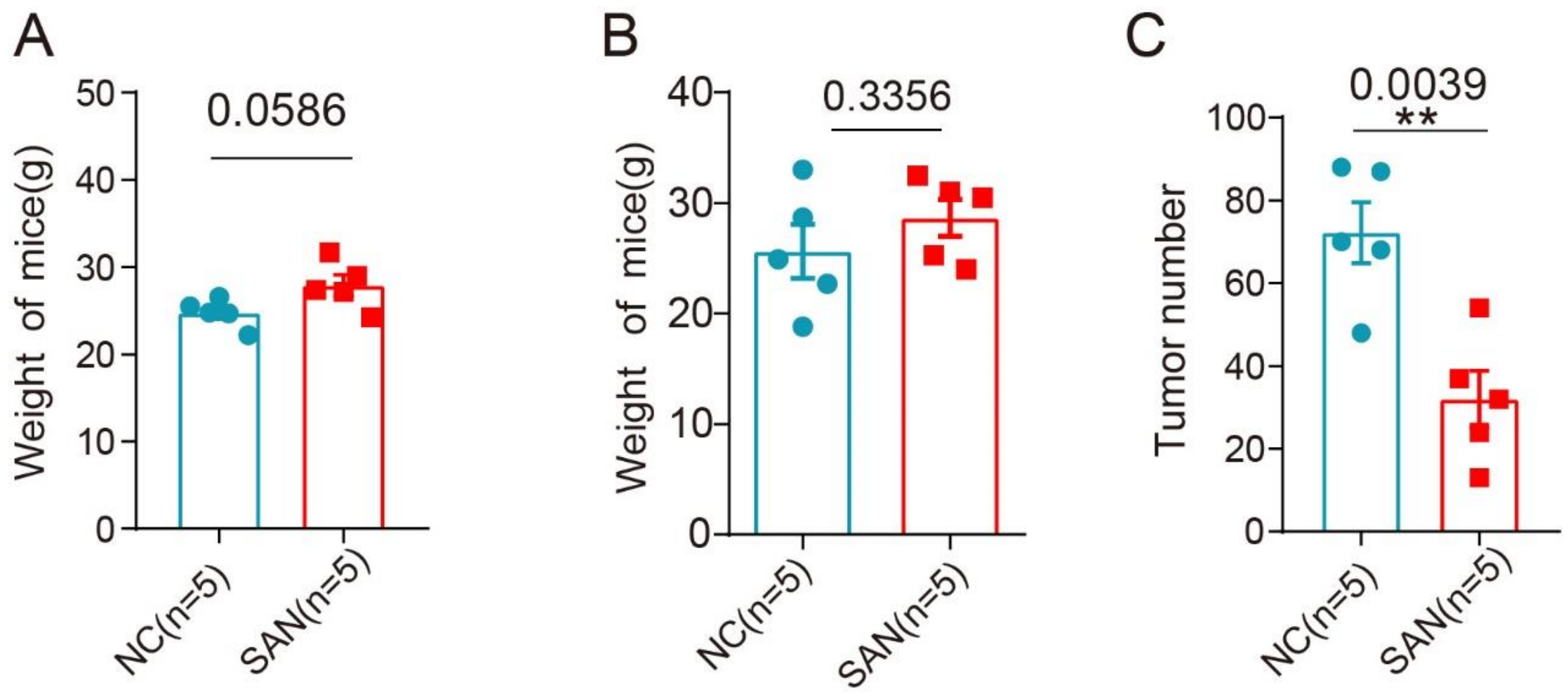

Supplementary Figure 9. Sanguinarine (SAN) inhibits lung cancer progression *in vivo*. (A) Analysis of mouse body weight in the subcutaneous tumor model (n = 5). (B) Weight analysis of the *Kras* $^{LSL\text{-}G12D/+}$*; Trp53*$^{fl/fl}$ mouse model (n = 5). (C) Analysis of tumor counts in the *Kras* $^{LSL\text{-}G12D/+}$*; Trp53*$^{fl/fl}$ mouse model (n = 5). Statistical significance was determined using Student's *t*-test. *, $P < 0.05$; n.s., not significant.

Supplementary Figure 10

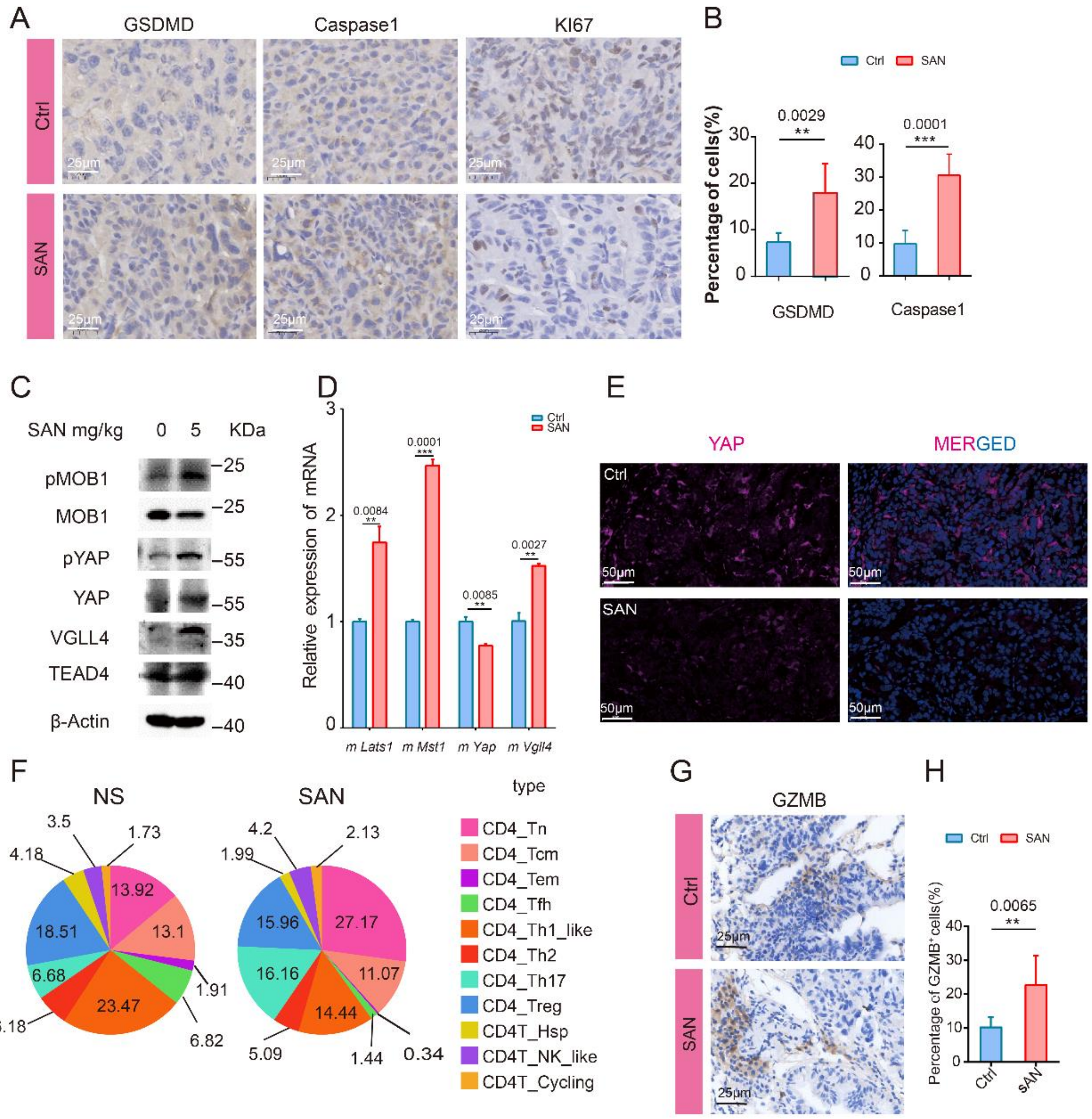

Supplementary Figure 10. Sanguinarine (SAN) activates the Hippo pathway and promotes cell pyroptosis *in vivo*. (A and B) Immunohistochemistry of gasdermin D (GSDMD), caspase-1, and Ki-67 in mouse lung cancer tissues treated with SAN or normal saline. Scale bars, 25 μm. (C) Western blotting analysis of subcutaneous tumor tissue in mice treated with SAN or normal saline. (D) RT-qPCR analysis of subcutaneous tumor tissue of mice treated with SAN or normal saline. **(**E) Immunofluorescence staining analysis of YAP in mouse lung cancer tissues treated with SAN or normal saline. Scale bars, 50 μm. (F) Analysis of the proportions of different cell types in the lung tissues of mice with lung cancer treated with SAN or normal saline. (G) Immunohistochemistry staining of GZMB in mouse lung cancer tissues treated with

SAN or normal saline. Scale bars, 25 μm. (H) Statistical analysis of the percentage of GZMB-positive cells in mouse tumor tissues following administration of SAN versus physiological saline. Statistical significance was determined using Student's t-test. *, $P < 0.05$; n.s., not significant.

Supplementary Figure 11

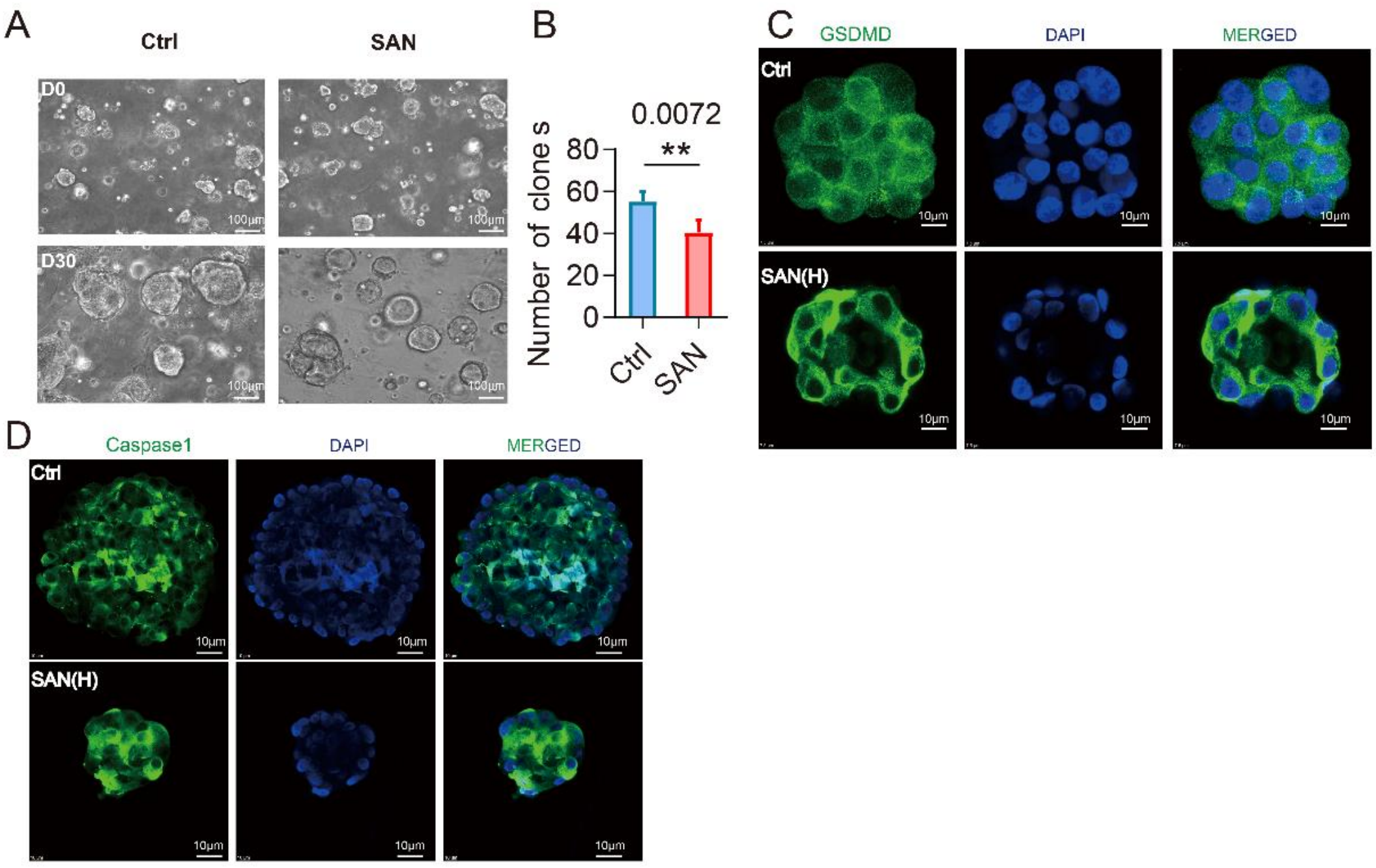

Supplementary Figure 11. Sanguinarine (SAN) inhibits the formation of lung cancer organoids by promoting pyroptosis. (A) Representative images of human-derived organoids from the control and SAN-treated groups at day 0 and day 30 of culture. Scale bars, 100 µm. (B) Statistical analysis of SAN-mediated inhibition of clonogenicity in human lung cancer organoids. Statistical significance was determined using Student's *t*-test. *, $P < 0.05$; n.s., not significant. (C and D) Immunofluorescence staining of caspase-1 and gasdermin D (GSDMD) in human-derived lung cancer organoids treated with SAN (3 µM) or vehicle (DMSO). Scale bars, 10 µm.

Materials and Methods

Cell lines and tissue samples

A549 (ATCC-CRM-CCL-185) cells were cultured in high-glucose DMEM (Gibco). H1299 (ATCC-CRL-5803), H1437(ATCC-CRL-5872) and PC9 (RRID: CVCL_D7FJ) cells were cultured in RPMI-1640 medium (Gibco). BEAS-2B (ATCC-CRL-3588) cells were cultured in Airway Epithelial Cell Basal Medium (Gibco). All cells were cultured under standard conditions, with 10% FBS (ExCell Bio). The cells were placed in a 37°C, 5% CO_2 incubator. Cells in the exponential growth phase were used for the experiments. Human tumor tissues were collected from Fudan University Shanghai Cancer Center. Ethics approval for this study was obtained from Medical Ethics Committee of Affiliated Hospital of Fudan University at Affiliated Hospital of Fudan University (approval number: 2301268-3). All participants were provided with information regarding the study and gave their written informed consent prior to participation. This study was conducted in compliance with the Declaration of Helsinki and all applicable ethical guidelines.

Kras $^{LSL\text{-}G12D/+}$; Trp53$^{fl/fl}$ mouse models

Kras $^{LSL\text{-}G12D/+}$*; Trp53*$^{fl/fl}$ mice were infected by intranasally delivering a 63.5 μL suspension of 2 × 10^7 PFU Adeno-Cre. After 2 weeks, the mice were intraperitoneally injected with sodium chloride solution or SAN every 3 days for the remainder of the experiment. Tumor sizes and numbers in the lungs were measured by IHC after 2 months. Ethics approval for this study was obtained from Laboratory Animal Center Fudan University at Fudan University (approval number: 20240229-003).

Lung cancer organoid culture

Surgically resected primary tumors were collected from patients with LUAD. For the cultivation of organoids, the tumor tissues are cut into 5-millimeter diameter blocks and washed with PBS. Subsequently, at 37°C, the tumor blocks are treated gently with a shaking motion using Advanced DMEMF12 (GIBCO) medium and collagenase XI

(Sigma) for 40 minutes. Cells were counted and resuspended in growth factor-reduced basement membrane extract (BME, R&D), plated in 24-well tissue culture plates as BME domes and maintained at 37°C with 5% CO_2 with medium overlaying the BME dome. Organoid growth was monitored weekly.

CCK-8 and colony formation assays

For the CCK-8 and cell aggregation experiments, human lung cancer cell lines (A549 and H1299) as well as normal lung epithelial cells (BEAS-2B) were plated into 96-well plates at a seeding density of 1×10^4 cells/well. Viability was quantified spectrophotometrically (OD450) after incubating cells with CCK-8 reagent (Yeasen Biotechnology) per the manufacturer's guidelines. Clonogenic potential was assessed after fixing (4% paraformaldehyde [PFA]) and staining (crystal violet) 2-week cultures in six-well plates, with colonies counted using ImageJ software. For the soft agar colony formation assay, a 0.8% agarose solution was used as the bottom layer, and a 0.5% agarose solution containing 1×10^4 cells/mL A549 cells and the corresponding concentrations of SAN was used as the upper layer. After 30 days of culture, colonies with a diameter exceeding 50 μm were counted.

Migration assays

A549 and H1299 cells were plated in six-well plates and grown to 80% confluence. A linear scratch was made uniformly across the monolayer using a 10-μL pipette tip. Cells were then cultured in 5% FBS medium containing SAN (0, 1.5, or 3 μM) or the vehicle control. Images of the scratches were taken at 0, 12, and 24 h post-wounding under an inverted microscope, and the percentage of wound closure was calculated using ImageJ with the formula: [(wound width at 0 h − wound width at indicated time) / wound width at 0 h] × 100%. In the transwell chamber assay, 1×10^5 A549 or H1299 cells suspended in serum-free medium were added to the upper inserts, and the lower chambers were filled with 10% FBS medium containing SAN. Following a 48-h incubation, cells remaining on the upper membrane surface were wiped off. The

migrated cells on the lower side were fixed with 4% PFA for 20 min, stained with crystal violet, and counted under a microscope.

Immunoblotting

Cells and tumor tissues were lysed in RIPA buffer on ice to obtain total protein extracts. The lysates were subjected to SDS-PAGE (10%) to separate proteins according to size, followed by electroblotting onto PVDF membranes. The membranes were then blocked with 5% milk for 1 h and probed with primary antibodies diluted in blocking buffer at 4°C overnight. Thereafter, the blots were incubated with HRP-linked secondary antibodies for 2 h. Then, the bands on the membrane were visualized under a chemiluminescence imaging system using an enhanced chemiluminescence kit. GAPDH and β-actin were used as loading controls. Western blots were probed with mouse anti-GAPDH (ABclonal; RRID: AC003), mouse anti-β-actin (ABclonal; RRID: AC004), rabbit anti-LATS1 (Cell Signaling Technology; RRID: 3477S), rabbit anti-pLATS1 (Cell Signaling Technology; RRID: 9157S), rabbit anti-MOB1 (Cell Signaling Technology; RRID: 13730S), rabbit anti-p-MOB1 (Cell Signaling Technology; RRID: 8699S), rabbit anti-YAP (Cell Signaling Technology; RRID: 14074S), rabbit anti-p-YAP (Cell Signaling Technology; RRID: 4911S), rabbit anti-caspase-1 (absin; RRID: abs128793), rabbit anti-cleaved caspase-1 (ABclonal; RRID: A23429), rabbit anti-GSDMD (Cell Signaling Technology; RRID: 39754T), or rabbit anti- cleaved GSDMD antibody (ABclonal; RRID: A24059).

IHC and IF staining

Tumor tissues from Kras $^{LSL-G12D/+}$; Trp53$^{fl/fl}$ mice were fixed in 4% PFA, embedded in paraffin, and sectioned. The sections were deparaffinized, rehydrated, and subjected to antigen retrieval in citrate buffer at 95°C for 30 minutes. Incubating with goat serum for 1 hour, the sections were incubated with the primary antibodies against Ki-67, GSDMD, GSDMD-N, caspase-1, cleaved-caspase-1, caspase-3, and YAP (1:200 dilution). After incubation with enzyme-conjugated secondary antibody, the signals

were visualized using 3,3’-diaminobenzidine substrate. Following counterstaining with hematoxylin, dehydration, clearing, and mounting, the sections were imaged under a microscope, and positive staining was quantified using ImageJ software.

Plasmid construction and transfection

To validate the regulatory role of Hippo pathway key molecules (YAP and LATS1) in SAN induced lung cancer cell pyroptosis, recombinant plasmids were constructed. The full-length coding sequence of human *YAP* (GenBank: NM_001130145.1) and human *LATS1* (GenBank: NM_ 001270519.2) were amplified from A549 cell cDNA and cloned into a pcDNA3.1-HA (RRID: Addgene_128034) vector to generate overexpression plasmids. All the target fragments and vectors were ligated with T4 DNA ligase, and then transfected into *Escherichia coli* DH5α. The positive clones were verified by Sanger sequencing. We constructed the LATS1 triple-mutant plasmid via homologous recombination. Specifically, DNA fragments carrying the mutation sites and flanked by homology arms were first generated. The vector was then linearized by digestion with appropriate restriction enzymes. Subsequently, the mutated DNA fragments and the linearized vector were subjected to homologous recombination using recombinase. Finally, the complete plasmids with correct site mutations were verified by Sanger sequencing. High-purity endotoxin-free plasmids (OD260/OD280: 1.8–2.0) were prepared using EndoFree Mini Plasmid kits. The overexpression/knockdown efficiency was confirmed by qPCR and immunoblotting after transfecting KP1 and H1299 cells.

RNA isolation and RT-qPCR

RNA was extracted from cells using TRIzol, and cDNA synthesis was performed with PrimeScript™ RT Master Mix. Real-time PCR was conducted using TB Green™ Premix Ex Taq™ II (Takara). Gene expression levels were quantified relative to β-actin via the $2^{-\Delta\Delta Ct}$ method[1].

Receptor model and molecular docking (MOE)

A template-based homology model of human LATS1 (residues 662-1,062; UniProt accession O95835) was retrieved from the "Structure" section of UniProt as a SWISS-MODEL entry (model identifier: O95835_662-1062:sn23.1.A, chain A). The model was prepared by adding hydrogen atoms and assigning protonation states under physiological conditions, followed by restrained energy minimization to alleviate local steric clashes while preserving the overall fold. Molecular docking of SAN was performed using MOE with the modeled LATS1 structure as the receptor. The binding site was defined according to the predicted pocket surrounding the key interacting residues observed in the docking pose, and the top-ranked pose was selected according to the docking score and reasonable interaction geometry. As the receptor is a homology model, the interaction pattern is described as predicted docking.

Statistical analysis

All data in this study are represented as the mean ± mean standard error. GraphPad Prism (version 8.3.0) and Image J were used to analyze the results and draw charts. Between-group differences were analyzed by Student's t-test. One-way ANOVA with Bonferroni correction was used for normally distributed data when comparing more than two groups, whereas the Kruskal-Wallis test with Dunn's post-hoc test was employed for non-normal data. Statistical significance was defined as $P < 0.05$.

1. Livak KJ, Schmittgen TD. Analysis of relative gene expression data using real-time quantitative PCR and the 2(-Delta Delta C(T)) Method. Methods. 2001; 25: 402-8.
